# Boundary-directed epitaxy of block copolymers

**DOI:** 10.1038/s41467-020-17938-3

**Published:** 2020-08-19

**Authors:** Robert M. Jacobberger, Vikram Thapar, Guang-Peng Wu, Tzu-Hsuan Chang, Vivek Saraswat, Austin J. Way, Katherine R. Jinkins, Zhenqiang Ma, Paul F. Nealey, Su-Mi Hur, Shisheng Xiong, Michael S. Arnold

**Affiliations:** 1 Department of Materials Science and Engineering, University of Wisconsin-Madison, Madison, WI 53706 USA; 2 School of Polymer Science and Engineering, Chonnam National University, Gwangju, 61186 South Korea; 3 Pritzker School of Molecular Engineering, University of Chicago, Chicago, IL 60637 USA; 4 MOE Key Laboratory of Macromolecular Synthesis and Functionalization, and Key Laboratory of Adsorption and Separation Materials and Technologies of Zhejiang Province, Department of Polymer Science and Engineering, Zhejiang University, Hangzhou, 310027 China; 5 Department of Electrical and Computer Engineering, University of Wisconsin-Madison, Madison, WI 53706 USA; 6 Department of Electrical Engineering, National Taiwan University, Taipei, 10617 Taiwan; 7 Department of Engineering Physics, University of Wisconsin-Madison, Madison, WI 53706 USA; 8 School of Information Science and Technology, Fudan University, Shanghai, 200433 China

**Keywords:** Polymers, Electronic properties and devices, Molecular self-assembly, Surface patterning

## Abstract

Directed self-assembly of block copolymers (BCPs) enables nanofabrication at sub-10 nm dimensions, beyond the resolution of conventional lithography. However, directing the position, orientation, and long-range lateral order of BCP domains to produce technologically-useful patterns is a challenge. Here, we present a promising approach to direct assembly using spatial boundaries between planar, low-resolution regions on a surface with different composition. Pairs of boundaries are formed at the edges of isolated stripes on a background substrate. Vertical lamellae nucleate at and are pinned by chemical contrast at each stripe/substrate boundary, align parallel to boundaries, selectively propagate from boundaries into stripe interiors (whereas horizontal lamellae form on the background), and register to wide stripes to multiply the feature density. Ordered BCP line arrays with half-pitch of 6.4 nm are demonstrated on stripes >80 nm wide. Boundary-directed epitaxy provides an attractive path towards assembling, creating, and lithographically defining materials on sub-10 nm scales.

## Abstract (web summary)

Directing the position, orientation, and long-range lateral order of block copolymer domains to produce technologically-useful, sublithographic patterns is a challenge. Here, the authors present a promising approach to overcome the challenge by directing assembly using spatial boundaries between planar, low-resolution regions on a surface with different composition.

## Introduction

Directed self-assembly of block copolymers (BCPs) provides a scalable route for rationally fabricating large-area patterns with dense nanoscale features at precise locations on a surface^1–4^. Directed assembly of BCP domains oriented vertically to the substrate is particularly important because vertical domains can be utilized to produce nanostructures for logic gates^5,6^, data storage^6,7^, electrical contacts^8^, optical devices^9^, sensors^10^, and separation membranes^11,12^, and to provide a template for materials synthesis^13–15^.

Predominantly, two techniques—graphoepitaxy^16^ and chemoepitaxy^17–19^—have been used to direct the assembly of vertical BCP domains into organized, useful patterns. Graphoepitaxy uses topographic features, such as trenches, whereas chemoepitaxy uses periodic chemical patterns to direct assembly of BCPs with controlled domain position, orientation, and lateral order. In both techniques, assembly of vertical lamellae can be controlled by tailoring template dimensions^20–23^ as well as interfacial interactions between the template surfaces and polymer blocks^20,24^, and feature density can be multiplied compared to the template dimensions to enhance resolution beyond the limits of conventional lithography^25,26^.

While the achievements of graphoepitaxy and chemoepitaxy (as well as hybrid processes adapting aspects of both techniques^27–29^) have been extensive^1–4^, directed assembly via these methods faces several challenges. For example, graphoepitaxy is sensitive to underfilling and overfilling of BCP in the trenches^30,31^, and requires fabrication of topographic structures that interfere with subsequent processing if the structures cannot be removed^32^. On the other hand, chemoepitaxy requires the formation of narrow guiding features that have widths of 0.5–1.5 times the BCP domain spacing (*L*_0_), where a width of 0.5*L*_0_ is ideal^21–23^. Therefore, to achieve BCP assemblies with sub-10 nm domains via chemoepitaxy, it is advantageous to use sub-10 nm guiding features; however, such features are difficult to fabricate. Moreover, at sub-10 nm scales, line-edge and surface roughness of the guiding features can become significant compared to *L*_0_, disrupting BCP registration^33^. While alternative chemical patterns with larger guiding features up to 3.5*L*_0_ in width have been used to direct assembly of vertical lamellae, the lamellae are not spatially registered and complex geometries (e.g., T-junctions, jogs, and bends) cannot be directed to assemble^34–37^.

Here, we present a promising paradigm to direct the assembly of BCPs using spatial boundaries between regions with different surface composition. The boundaries can be defined between two relatively low-resolution, planar features, circumventing the need for topographic structures or high-resolution chemical patterns to direct assembly. This technique, termed boundary-directed epitaxy, is demonstrated using a model template comprised of isolated graphene stripes on a Ge substrate. The graphene stripes are atomically thin and smooth, presenting minimal topography, and have atomically faceted edges, resulting in abrupt transitions in surface composition at each stripe edge.

## Results and discussion

### Boundary-directed epitaxy of block copolymers

**Fig. 1:**
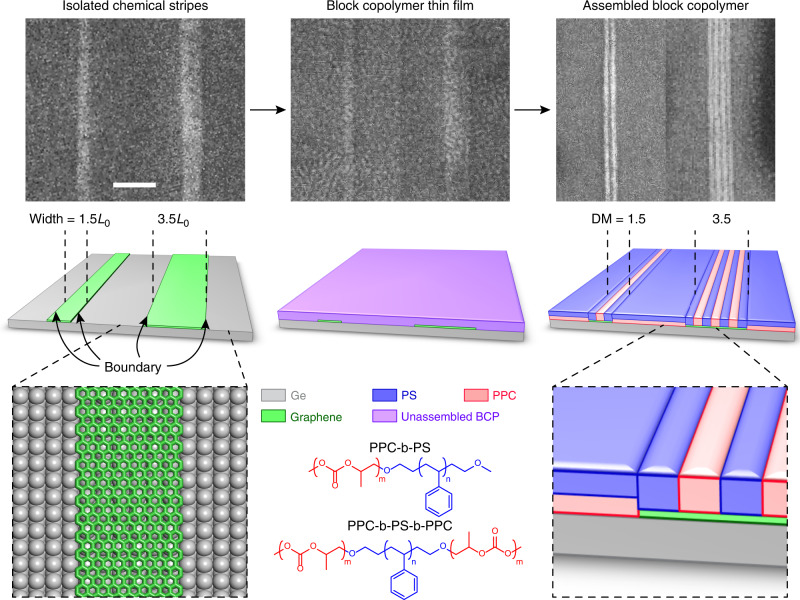
Boundary-directed epitaxy of block copolymers. Scanning electron microscopy (SEM) images (top) and corresponding schematic diagrams (middle row) after growth of graphene stripes on Ge via CVD (left), spin-coating of PPC-b-PS-b-PPC (*L*_0_ of 12.8 nm) thin films (middle column), and thermal annealing (right). Assembly on stripes with widths of 1.5 and 3.5*L*_0_, corresponding to density multiplication factors (DM) of 1.5 and 3.5, respectively. Vertical PS lamellae border both edges of the stripes, as determined from the SEM images (PS is bright and PPC is dark)^44^. Scale bar is 100 nm. Schematic diagrams (bottom) of graphene stripes on a Ge(001) surface (left), BCP chemical structures (middle column), and transition region between vertical and horizontal BCP lamellae at the stripe/substrate boundary (right). The graphene stripe thickness is exaggerated so that the stripes can be visualized more easily.

Figure 1 shows the process flow for boundary-directed epitaxy. The chemical contrast at each stripe/substrate boundary nucleates and pins the formation of vertical lamellae along the stripe edges. Vertical lamellae then propagate into the interior of each stripe, resulting in registered vertical line arrays that selectively form on stripes, self-align parallel to the boundaries, and register to wide and incommensurate stripes to multiply the feature density. In contrast, horizontal lamellae form on the background; thus, the lamellar orientation is sharply modulated at the stripe edges.

Simulations of assembly of generic BCPs on generic template surfaces show boundary-directed epitaxy is viable over a relatively wide window of surface and interfacial energies, provided appropriate chemical contrast at the stripe/substrate boundary is achieved, demonstrating that this is a general approach that can be extended to various stripe/substrate/BCP combinations. However, graphene, investigated here, is an attractive stripe material because it is atomically thin and smooth, impermeable against swelling, and stable under a wide range of chemical and thermal processing conditions. Moreover, graphene can be integrated directly onto large-area, conventional semiconductor wafers with atomic-layer thickness control via chemical vapor deposition (CVD).

To fabricate the chemical pattern, graphene stripes are synthesized directly on Ge(001) wafers via CVD by decomposing CH_4_ at 910 °C in a flow of H_2_ and Ar^38^. This bottom-up synthesis yields graphene stripes with tunable width from virtually 0 to 100s of nanometers by adjusting the growth time, with high-aspect ratio due to the anisotropic nature of growth, with single-atom thickness of 3 Å due to a self-limiting growth mechanism, and with atomically sharp edges due to faceting of the graphene crystals (with edge roughness <5 Å over edge lengths >10 nm)^38–40^. Furthermore, the surfaces of graphene and Ge are relatively pristine and uncontaminated (Supplementary Fig. 1)^41,42^, providing a clean template for assembly. Two CVD approaches are used here. First, CVD on bare Ge(001) is used to produce graphene stripes that are polydisperse in width (0–100 nm) to study the effect of stripe width on assembly. Second, CVD employing graphene seeds that are lithographically positioned on Ge(001) is used to produce regular arrays of graphene stripes with controlled placement, unidirectional alignment, and reduced polydispersity^43^. Ultimately, it should also be possible to grow continuous films of graphene and then arbitrarily pattern graphene into stripes via lithography.

Boundary-directed epitaxy is demonstrated using lamellae-forming poly(propylene carbonate)-block-polystyrene (PPC-b-PS) di-BCP^44^ with *L*_0_ of 16.8 nm and PPC-b-PS-b-PPC tri-BCPs^45^ with *L*_0_ of 12.8 and 14.5 nm (Supplementary Table 1). The BCPs are deposited onto the graphene/Ge template via spin-coating, yielding BCP films that initially lack long-range order and have thickness of ~*L*_0_. The graphene and Ge surfaces are chemically preferential to PS and PPC, respectively (Supplementary Figs. 2–8). Following spin-coating, the samples are annealed at 160 °C for 10 min to microphase separate and self-assemble the BCP chains. Such annealing on continuous graphene films or bare Ge substrates leads to the formation of horizontal lamellae because of their chemical preferences (Supplementary Fig. 8). However, due to the presence of the boundary when graphene and Ge are laterally adjacent, vertical and horizontal lamellae form on graphene and Ge, respectively, and domain orientation is sharply modulated directly at the stripe/substrate boundary. This modulation at the stripe/substrate boundary indicates that the epitaxy of the BCP domains is boundary-directed.

**Fig. 2:**
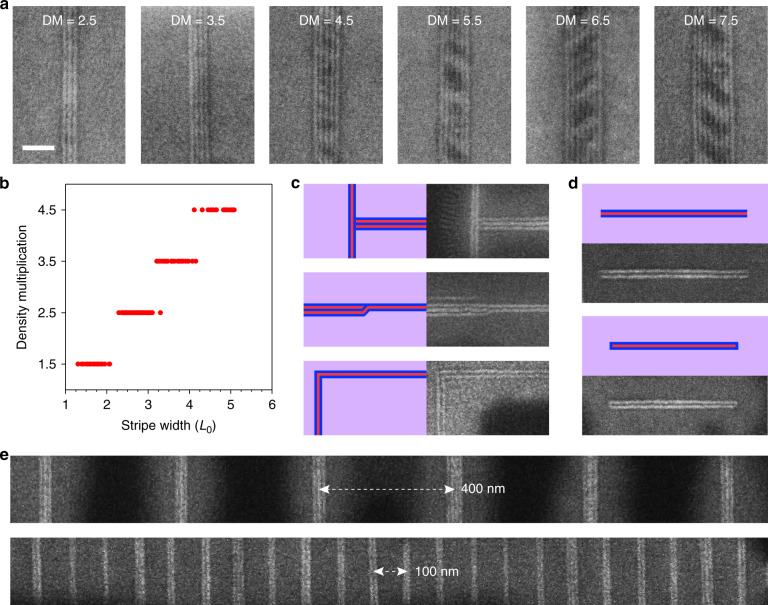
Density multiplication and pattern formation. **a** SEM images of PPC-b-PS (*L*_0_ of 16.8 nm) after assembly on stripes with widths of ~2.5, 3.5, 4.5, 5.5, 6.5, and 7.5*L*_0_ (left to right). **b** Plot of density multiplication factor (DM) versus stripe width for PPC-b-PS-b-PPC (*L*_0_ of 12.8). **c** PPC-b-PS-b-PPC (*L*_0_ of 12.8 nm) assembled into various patterns, including T-junctions (top), jogs (middle row), and 90° bends (bottom). **d** PPC-b-PS-b-PPC (*L*_0_ of 12.8 nm) assembled to form isolated arrays of three lines. On some stripes, vertical PS lamellae directly border both the long and short stripe edges (bottom), whereas on other stripes (most often, when width is ~1.5*L*_0_), vertical PS lamellae only directly border the long stripe edges (top). Vertical PS and PPC lamellae are blue and red, respectively, and horizontal lamellae are purple. Scale bar for **a**, **c**, **d** (shown in **a**) is 100 nm. **e** SEM images of PPC-b-PS-b-PPC (*L*_0_ of 12.8 nm) assembled on seeded stripe arrays with superstructure pitch of 400 (top) and 100 nm (bottom).

The vertical lamellae that selectively form on each graphene stripe self-align parallel to and register along the stripe’s long axis. Numerous vertical lamellae can span the width of each stripe to produce periodic line arrays, even on stripes much wider than *L*_0_, thereby multiplying the feature density compared to stripe width (Supplementary Figs. 9 and 10). Vertical PS (rather than PPC) lamellae border and register to both long edges of the stripes, resulting in an odd number of lines and density multiplication of *n* + ½, where *n* is a positive integer. For example, stripe widths of 1.5, 2.5, 3.5, and 4.5*L*_0_ result in 3, 5, 7, and 9 lines, respectively (Fig. 2a). PS-b-PPC with *L*_0_ of 16.8 nm and PPC-b-PS-b-PPC with *L*_0_ of 12.8 and 14.5 nm can assemble with density multiplication factors as large as 8.5, 9.5, and 15.5, respectively (Supplementary Fig. 11), enabling epitaxy of sub-10 nm BCP domains on templates with a smallest defined dimension of ~100 nm. The density multiplication factor depends on the anneal time and temperature, as discussed in more detail below and in Supplementary Table 3. Density multiplication is observed on stripes as long as 1.5 μm (Supplementary Fig. 12), but this length is only limited by variation in the stripe width and can be increased in future work by improving stripe fabrication.

Boundary-directed epitaxy is also achieved when the stripe width is incommensurate with (*n* + ½)*L*_0_. In fact, self-aligned vertical lamellae of PPC-b-PS-b-PPC (*L*_0_ of 12.8) assemble on stripes of every possible width spanning 15–100 nm (Fig. 2b). In instances where multiple graphene stripes join to form intersections, turns, and constrictions, complex BCP patterns including T-junctions, jogs, and 60, 90, and 120° bends assemble (Fig. 2c and Supplementary Fig. 13). Such features are essential for fabricating integrated circuits^46,47^. Particularly noteworthy is the ability to fabricate isolated line arrays (Fig. 2d and Supplementary Fig. 13) and their superstructures (Fig. 2e and Supplementary Fig. 10). Assembly even proceeds when individual neighboring stripes are as close as 0.5*L*_0_ (i.e., 5–10 nm) (Supplementary Fig. 14); therefore, densely packed superstructures should ultimately be viable.

**Fig. 3:**
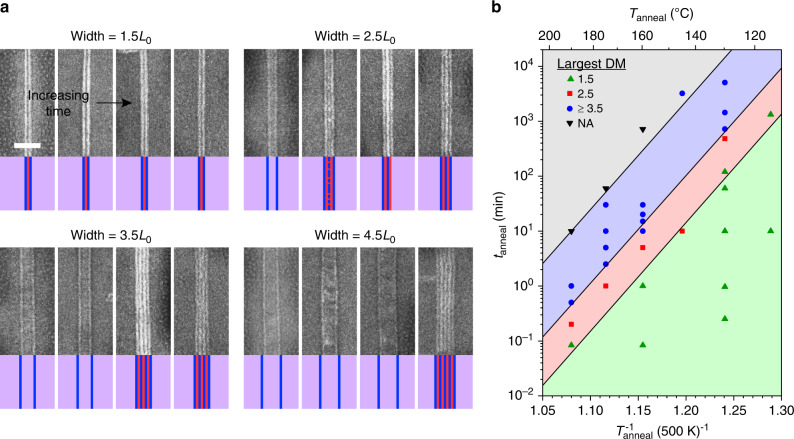
Effect of anneal time, anneal temperature, and stripe width. **a** SEM images and schematic diagrams corresponding to PPC-b-PS-b-PPC (*L*_0_ of 12.8 nm) morphology after assembly at 130 °C for 1, 10, 1440, and 5040 min (left to right) on stripes with width of ~1.5, 2.5, 3.5, and 4.5*L*_0_. Vertical PS and PPC lamellae are blue and red, respectively, and horizontal lamellae are purple. Scale bar is 100 nm. **b** Plot of the largest density multiplication factor (DM) achieved as a function of anneal time (*t*_anneal_) and anneal temperature (*T*_anneal_). Each data point represents a unique assembly experiment, in which the largest density multiplication is 1.5 (green upward triangles), 2.5 (red squares), ≥3.5 (blue circles), or not applicable (NA) because BCP did not assemble (black downward triangles). Black lines and shading are shown only to highlight approximate transitions.

**Fig. 4:**
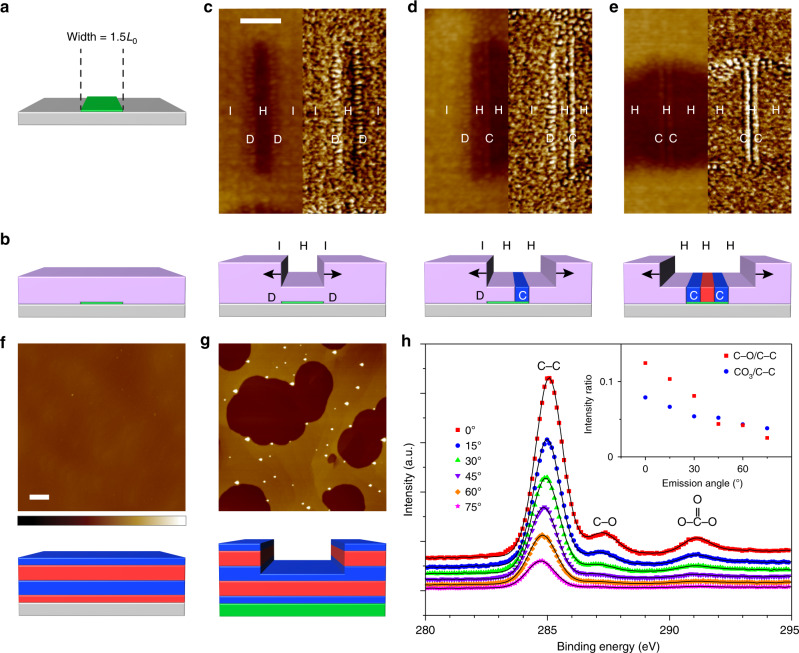
Effect of hole and island formation. Schematic diagrams (**a**–**e**) and AFM height (left) and corresponding phase (right) images (**c**–**e**) of PPC-b-PS-b-PPC (*L*_0_ of 12.8 nm) morphological evolution during assembly. Holes (H) and islands (I) within the BCP film topography as well as discontinuous domains on the substrate (D) and continuous vertical lamellae on the stripe (C) at the stripe/substrate boundary are indicated. Scale bar for **c**–**e** (shown in **c**) is 100 nm. Graphene, Ge, vertical PS lamellae, vertical PPC lamellae, and horizontal lamellae are green, gray, blue, red, and purple, respectively. A detailed description of the morphology of the horizontal lamellae is provided in Fig. 5, below. AFM topographic maps of PPC-b-PS-b-PPC (*L*_0_ of 12.8 nm) with initial thickness of ~1.5*L*_0_ after assembly on a bare Ge surface (**f**) and on a continuous graphene film (**g**). Schematic diagrams show the corresponding side-view of the BCP film morphology. Scale bar for **f**, **g** (shown in **f**) is 1 μm and height scale is 60 nm. Graphene, Ge, PS, and PPC are green, gray, blue, and red, respectively. **h** Angle-resolved XPS of the C1s spectrum of the film in **f** collected at emission angles of 0, 15, 30, 45, 60, and 75° (top spectrum to bottom spectrum). Inset plots the integrated intensity ratio of the C─O peak (red squares) and CO_3_ peak (blue circles) to the C─C peak versus emission angle. See Supplementary Figs. 2–8 and Supplementary Note 1 for additional detail. The graphene stripe thickness is exaggerated so that the stripes can be visualized more easily.

Boundary-directed epitaxy is successful on graphene stripes with armchair edges (i.e., C─C bonds are parallel to the stripe long-axis) on relatively unoxidized Ge(001) surfaces (Figs. 1–4), but also on graphene stripes with different crystallographic edge orientations, Ge with different surfaces orientations, and Ge with varying degrees of oxidation (Supplementary Figs. 15–17). Thus, this mode of assembly is relatively robust with respect to the atomic structure and composition of the stripe and background.

While graphene has been studied as a template for directing BCP assembly^48^, the assembly phenomena and mechanisms reported here differ significantly from earlier work. Specifically, graphene has previously been used to direct assembly in conventional chemoepitaxy guiding schemes based on periodic arrays of narrow guiding features that match the size of the BCP domains (width of ~0.5*L*_0_). In contrast, here, assembly is driven by chemical contrast achieved at graphene stripe edges, without the need for high-resolution chemical features.

### Evolution of vertical lamellae and film topography

The kinetics of boundary-directed epitaxy show that vertical BCP lamellae first nucleate at the edges of each stripe, but only after a mass transfer event in which the BCP partially flows off each stripe and onto the substrate, thereby inducing spatial variation in film thickness. These phenomena are demonstrated in Figs. 3 and 4, in which the evolution of vertical lamellae and BCP film topography are investigated, respectively, using PPC-b-PS-b-PPC (*L*_0_ of 12.8 nm).

Evolution of vertical lamellae as a function of stripe width is characterized at a relatively low anneal temperature of 130 °C (Fig. 3a) to ensure slow kinetics so that assembly progression can be observed. Immediately after spin-coating, the BCP film lacks long-range order. However, vertical lamellae quickly form on graphene along each stripe edge in <1 min of annealing, regardless of stripe width. These rapidly forming domains self-align to each stripe/substrate boundary, where they remain pinned throughout assembly. On stripes wider than ~2.5*L*_0_, discontinuous dot-like domains form in the stripe interiors (Fig. 3a and Supplementary Fig. 18) and eventually coalesce to form new vertical lamellae with further annealing. These new domains self-align to the existing domains pinned at the edges; therefore, alignment of vertical lamellae during density multiplication propagates from the edges to the center of stripes. The rate of propagation decreases with increasing stripe width. For example, density multiplication is achieved after ~1, 480, 1440, and 5040 min on stripes with widths of ~1.5, 2.5, 3.5, and 4.5*L*_0_, respectively (Fig. 3a). This evolution is similar to that observed during graphoepitaxy, in which assembly first occurs at vertical trench sidewalls and then propagates to the trench center^49,50^ and density multiplication is more difficult across wider trenches^51^.

The assembly kinetics accelerate with increasing temperature (Fig. 3b, Supplementary Table 3, and Supplementary Figs. 19 and 20). For example, density multiplication ≥3.5 is achieved after annealing for ~1440, 10, 2.5, and 0.5 min at 130, 160, 175, and 190 °C, respectively. The kinetics exhibit an Arrhenius dependence^52^, indicating a thermally activated process with apparent activation energy of ~2 eV. Assembly is disrupted, however, if the anneal time is too long or temperature is too high, leading to degradation or dewetting of the BCP film.

Mass transport of the BCP is also an important aspect of the assembly process. After spin-coating, the BCP film covers the graphene stripes and Ge substrate with uniform thickness (Fig. 4a, b). However, upon annealing, the BCP partially flows off graphene and onto Ge, resulting in thinner regions (i.e., holes) on the stripes and thicker regions (i.e., islands) on the substrate (Fig. 4c). When holes have formed on the stripe but islands still directly border the stripe edges, alternating, discontinuous domains form on the substrate along the stripe edges (Fig. 4c, d). However, after the hole/island boundary has progressed past the stripe/substrate boundary, vertical lamellae nucleate on the stripe (Fig. 4c–e). When mass transfer reaches steady-state, the graphene stripes are located in holes (Supplementary Fig. 21), and the BCP film thickness in holes and islands is ~0.5 and 1.5*L*_0_, respectively (Supplementary Fig. 22).

### Monte Carlo (MC) simulations of boundary-directed epitaxy

MC simulations are conducted to further understand the assembly mechanism and elucidate the assembly evolution with higher spatial and temporal resolution than in Figs. 3 and 4. Assembly of a generic A-b-B-b-A BCP (where A and B are different blocks) on a generic stripe/substrate system is modeled using a theoretically informed coarse-grained bead-spring model with a generalized energy functional (see Methods and Supplementary Note 2 for details). The simulated stripe, substrate, A, and B correspond to graphene, Ge, PPC, and PS, respectively, in experiments. The interaction strengths of the stripe and background with each polymer block are adjusted to reproduce the experimental preferentiality of graphene to PS and Ge to PPC, determined from atomic force microscopy (AFM) of the hole/island topography on continuous graphene films and bare Ge surfaces (Fig. 4f, g and Supplementary Figs. 2–8). The surface energy of each polymer block is adjusted by varying its incompressibility to reproduce the experimental preferential wetting of the free surface of the BCP film by PS, determined from angle-resolved X-ray photoelectron microscopy (XPS). Specifically, the C─O and CO_3_ XPS peaks are only characteristic of PPC (Supplementary Fig. 7). Thus, decreasing *I*_C─O_ and *I*_CO3_ with respect to *I*_C─C_ with increasing emission angle (Fig. 4h) indicates that PS preferentially wets the free surface (Fig. 4f, g). A disordered A-b-B-b-A film coated onto a stripe/substrate template is annealed by performing single-bead displacements, which captures the dynamic trajectories of BCP chains during assembly (see Methods and Supplementary Note 3 for details). Each MC cycle consists of *M* bead displacements, where *M* is the total number of beads in the system.

**Fig. 5:**
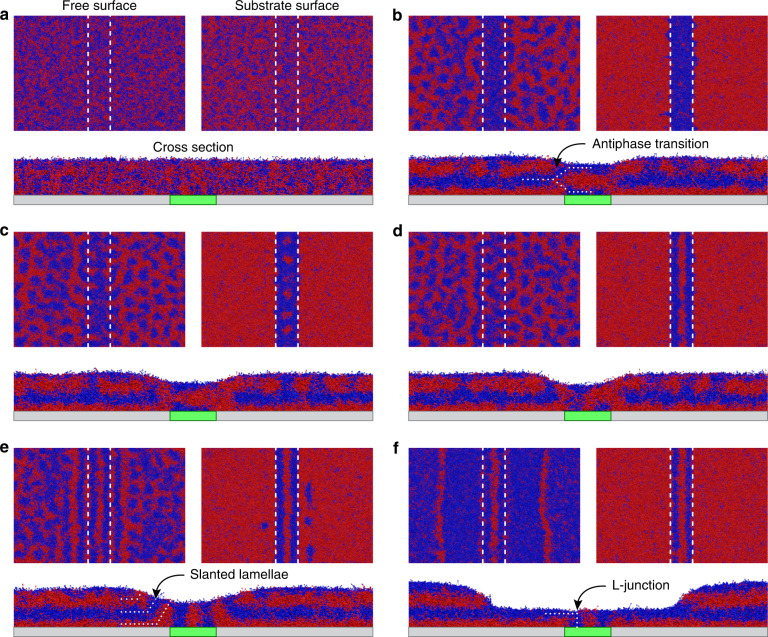
Monte Carlo simulations. Simulated BCP morphology at the free (top) surface (top left), at the substrate (bottom) surface (top right), and along a cross-section perpendicular to the stripe width (bottom) after 10^2^ (**a**), 6.*5* × 10^4^ (**b**), 1.25 × 10^5^ (**c**), 1.8 × 10^5^ (**d**), 3.0 × 10^5^ (**e**), and 2.15 × 10^6^ (**f**) MC cycles. The stripe, substrate, B block, and A block are green, gray, blue, and red, respectively. White dashed lines indicate the positions of the stripe/substrate boundaries. White dotted lines in **b**, **e**, **f** highlight an antiphase transition between horizontal lamellae, a set of slanted lamellae, and an L-junction, respectively. Since vertical B lamellae and horizontal B lamellae connect via L-junctions along the stripe edge at the free surface, it is difficult to differentiate these two domains in the top view in **f**. In contrast, in SEM images of the free surface (e.g., Figs. 1–3), vertical PS lamellae on the stripe display different contrast than horizontal PS lamellae on the substrate because the former domains are through-film while the latter domains are on top of at least one horizontal PPC domain.

When the appropriate range of interaction strengths, incompressibilities, and film thicknesses are used (see Supplementary Note 5 and Supplementary Table 4 for details), the evolution of the BCP morphology (Fig. 5) matches that observed experimentally (Figs. 3 and 4). The intermediate stages of assembly of an A-b-B-b-A film with relaxed thickness of 1.15*L*_0_ on a stripe with width of 1.5*L*_0_ are shown in Fig. 5. Upon annealing the initially disordered BCP film (Fig. 5a), the BCP reorganizes so that B and A preferentially wet the stripe and substrate, respectively, forming horizontal lamellae in which B and A are out of phase by 0.5*L*_0_ at each stripe/substrate boundary (Fig. 5b, c). Connectivity of the B and A domains across the antiphase transition is maintained via formation of curved, alternating B–rich and A–rich structures, resembling the alternating, discontinuous domains observed experimentally in Fig. 4c, d. These connected structures enable diffusion of the BCP chains and rearrangement of the BCP film topography. The BCP flows off the stripes, forming holes, and onto the substrate, forming islands, so that the B block can preferentially wet the free surface of the BCP film above the substrate. Later in the assembly process (Fig. 5f), the film thickness on the substrate becomes commensurate with 0.5*L*_0_ and 1.5*L*_0_ to form A-B and A-B-A-B horizontal lamellae, respectively, in which B wets the free surface. Preferential wetting of the free surface by B is therefore an important driving force for the flow of BCP from stripe to substrate. The film thickness increases gradually between the holes and islands. In the sloped regions, the horizontal lamellae on the substrate curve upward and contact the free surface at the top of the BCP film (Fig. 5d, e). Similar slanted lamellae have been observed at step edges in di-BCP films^53^.

The slanted lamellae act as a template to convert the lamellar domains on the stripes from horizontal to vertical. Specifically, the bottommost A lamella on the substrate curves upward to form a through-film domain directly bordering the stripe edge. This slanted A lamella nucleates the formation of a vertical B lamella on the stripe at the stripe/substrate boundary, which subsequently induces the formation of a vertical A lamella at the stripe center. Similar evolution is observed when the stripe width is increased to 2.5*L*_0_ and 3.5*L*_0_, accompanied by the formation of additional vertical A and B lamellae in the stripe interior (Supplementary Fig. 23). Slanted lamellae therefore drive the nucleation of and pin vertical lamellae along the stripe edges, mimicking sidewalls in graphoepitaxy; here, however, the first vertical lamellae nucleate spontaneously to form at stripe/substrate boundaries without predefined topographic features.

As BCP chains continue to diffuse from the stripe to the substrate, the bottommost slanted A lamella on the substrate disconnects from the free surface as the bottommost B lamella on the substrate connects to the vertical B lamella at the stripe edge to form an L-junction, resulting in uniform film thickness at the stripe/substrate boundary. With further annealing, the holes enlarge as the hole/island boundaries continue to diffuse away from the stripe edges, the step height between holes and islands continues to increase to ~*L*_0_, and the horizontal lamellae on the substrate approach the commensurate thicknesses of 0.5*L*_0_ in holes and 1.5*L*_0_ in islands (Fig. 5f), matching the BCP morphology observed experimentally (Figs. 1, 3, and 4). Driving forces governing the evolution observed in Fig. 5 are discussed in more detail in Supplementary Note 4 and Supplementary Fig. 24.

### Robustness of boundary-directed epitaxy

The MC simulations indicate boundary-directed epitaxy is relatively robust with respect to the chemical contrast at the stripe/substrate boundaries, the surface tension of the BCP blocks, and the initial BCP film thickness (Supplementary Note 5 and Supplementary Figs. 24–27). For example, with all other simulation parameters constant, epitaxy is realized when the interaction strength between B and the stripe is varied by 10%, the incompressibility of A is varied by 15%, the initial BCP film thickness is varied from 0.95*L*_0_ to 1.3*L*_0_, and the step height at the stripe/substrate boundaries is varied from 0 to 3.4 Å (the thickness of monolayer graphene).

The relatively large window of chemical contrast in which epitaxy is observed will translate experimentally to realizing assembly using a variety of stripe/substrate/BCP materials combinations beyond the graphene/Ge/PS-b-PPC system. This robustness is already evidenced experimentally to some extent by the insensitivity of boundary-directed epitaxy to the degree of Ge oxidation (Supplementary Fig. 17). In contrast, larger variations in chemical contrast that alter the preferentiality of the stripe or background to the polymer blocks disrupt this mode of epitaxy. For example, boundary-directed epitaxy is not observed if the background is neutral to both blocks. This scenario is experimentally demonstrated by replacing PPC with poly(methyl methacrylate) (PMMA) and assembling PS-b-PMMA (*L*_0_ of 25 nm) on graphene stripes on Ge. In this case, the graphene stripes are still preferential to PS, but Ge is neutral^48^, resulting in assembly of small disordered grains of vertical lamellae on Ge and horizontal lamellae on graphene (Supplementary Fig. 28). This morphology is reproduced by MC simulations (Supplementary Fig. 29a, b). The complementary scenario of isolated neutral stripes on a preferential background has been experimentally examined in literature^34^ and results in formation of horizontal lamellae on the background and vertical lamellae on the stripes. However, the vertical lamellae do not align along or register to stripe edges. This morphology is also reproduced by MC simulations (Supplementary Fig. 29c, d). Other simulation conditions that disrupt boundary-directed epitaxy are summarized in Supplementary Notes 4 and 5 and Supplementary Figs. 24–27.

## Conclusion

Boundary-directed epitaxy is a promising paradigm for controlling the position, orientation, and lateral order of BCP domains into sub-10 nm patterns, beyond the resolution of conventional lithography. Unlike other approaches, assembly is directed by a one-dimensional boundary between regions on a surface with different composition. When the regions are isolated, horizontal lamellae form on both surfaces due to their chemical preferences. However, when the regions are adjacent, the chemical contrast at their boundary sharply modulates the BCP orientation from horizontal to vertical. The regions are planar and are at least several times larger than the BCP domain spacing, circumventing the need for topographic structures or sub-10 nm chemical features and relaxing processing requirements for directing assembly of sub-10 nm BCP patterns. Boundary-directed epitaxy is used to direct isolated line arrays, superstructures of isolated line arrays, T-junctions, jogs, and bends, which are particularly useful for fabricating electronic devices. The demonstrated assembly of PPC-b-PS and PPC-b-PS-b-PPC on graphene stripes on a Ge surface can itself enable production of arrays of self-aligned semiconducting graphene nanoribbons^54^ or Ge fins^55^ if pattern transfer to the underlying template is optimized^44^. More broadly, boundary-directed epitaxy is expected to be viable using various BCPs and stripe/substrate materials combinations—fabricated via a variety of additive and subtractive patterning approaches^22,35,56,57^—provided that proper preferentiality towards each block of the BCP is achieved. Boundary-directed epitaxy may therefore provide a simple, scalable route for assembling, creating, and lithographically defining materials on sub-10 nm scales.

## Methods

### Synthesis of graphene stripes on Ge substrates

Graphene stripes are deposited directly on Ge wafers via CVD^38^. Ge(001) (Wafer World, >50 Ω-cm), Ge(110) (MTI Corporation, >50 Ω-cm), Ge(001) with 6° miscut towards Ge[110] (University Wafer, 0.01–0.05 Ω cm, Ga dopants), and Ge(001) with 9° miscut towards Ge[110] (Wafer World, 0.4 Ω cm, Sb dopants) substrates are loaded into a horizontal furnace with a quartz tube inner diameter of 34 mm. The system is evacuated to <10^−2^ torr and then refilled to atmospheric pressure with a flow of Ar and H_2_. After annealing the substrates at 910 °C, CH_4_ is introduced to begin graphene growth. The furnace is slid away from the growth zone to terminate growth while maintaining the same atmosphere used during graphene synthesis.

### Block copolymer assembly

PPC-b-PS di-BCP and PPC-b-PS-b-PPC tri-BCPs are synthesized via in-situ chain-transfer polymerization^44,45^. During polymerization, telechelic PS homopolymers with hydroxyl end group(s) serve as chain-transfer agents (CTAs). After deprotonation, the end-capped hydroxyl groups initiate the alternating incorporation of propylene oxide (PO) and CO_2_ in the presence of the most common Salen cobalt complex, producing PPC/PS BCPs. After removal of a small amount of PPC homopolymer, PPC-b-PS or PPC-b-PS-b-PPC with nearly uniform molecular weight and narrow molecular weight distribution is obtained. The reactions are conducted at room temperature using a PO:CTA:cobalt molar ratio of 2000:20:1 and 2.0 MPa of CO_2_. The volume ratio of PPC to PS in the BCP products is controlled by tuning the reaction time. After growth of graphene stripes on Ge via CVD, a BCP film with thickness of ~*L*_0_ is spin-coated onto the template surface. The BCP film is thermally annealed on a hotplate in an inert environment (1 atm of N_2_, with <1 ppm H_2_O and <1 ppm O_2_) to microsegregate the BCP domains. Boundary-directed epitaxy is achieved by thermal annealing, without relatively complex solvent annealing^58^ or topcoats^59^.

### Characterization

Samples are imaged with SEM (Zeiss LEO 1530) using an in-lens detector. Surface morphology is characterized via AFM (Veeco MultiMode SPM) in tapping mode. Characterization of surface composition is performed using XPS (Thermo K-Alpha) with spot size of 400 μm and monochromatic Al Kα radiation (1486.7 eV). Spectra of the C1s and Ge2p_3/2_ peaks are acquired using analyzer pass energy of 50 eV (resolution of 0.1 eV) and collection time of 50 ms. XPS peaks are fit to a Voigt function (Lorentzian:Gaussian ratio of 3:7) using the Powell fitting algorithm, after subtracting a Shirley-like background. Static contact angles are measured using an optical contact angle system (DataPhysics OCA 15). Deionized, distilled H_2_O droplets (volume of 7 µL) are dispensed onto the surfaces and H_2_O contact angles are measured immediately after droplet formation.

### MC simulations

Simulations are conducted using a coarse-grained bead-spring model that is derived from the theoretically informed coarse-grained (TICG) method^60,61^ (see Supplementary Note 2 for additional details), which has been successfully applied to model the assembly of BCP thin films. The system consists of polymer chains represented by a discrete number of beads, *N*. The system Hamiltonian has three contributions: (1) bonded interactions between beads, (2) non-bonded interactions between beads, and (3) interactions of beads with the template, and the adjustable thermodynamic parameters describing the interaction terms are (1) incompatibility between blocks, *xN*, (2) incompressibility of the B and A blocks, *k*_B_*N* and *k*_A_*N*, respectively, and (3) interaction strength between the stripe and B, the stripe and A, the substrate and B, as well as the substrate and A, *λ*_B-stripe_*N*, *λ*_A-stripe_*N*, *λ*_B-substrate_*N*, and *λ*_A-substrate_*N*, respectively. Negative and positive *λ* values imply attraction and repulsion, respectively. The non-bonded interaction Hamiltonian is expressed as a functional expansion in terms of number densities up to the third order^62–64^, which allows the coexistence of dense and sparse (or vacuum) regions to enable prediction of phenomena at the non-flat free surface at the top of the BCP film.

The model described above is evolved using MC simulations (see Supplementary Note 3 for additional details). Each simulation is conducted for at least 2 × 10^6^ MC cycles. Each MC cycle consists of *M* bead displacement steps, where *M* is the total number of beads in the system. The lateral dimensions of the simulation box are 17.5*R*_e_ × 7.5*R*_e_ × 3.75*R*_e_ (*L*_0_ is ~1.25*R*_e_), where *R*_e_ is the mean-squared end-to-end distance for an isolated non-interacting chain. The template, which consists of an isolated chemical stripe on a background surface, is below the BCP film. The BCP film has an initial thickness of 1.25*R*_e_ and an initial chain density of 128*R*_e_^−3^. The system is annealed at a given set of adjustable parameters. The values of *xN*, *k*_B_*N*, *k*_A_*N*, *λ*_B-stripe_*N*, *λ*_A-stripe_*N*, *λ*_B-substrate_*N*, and *λ*_A-substrate_*N* used in Fig. 5 are 35, 100, 200, −2.35, −2.0, −2.0, and −2.5, respectively (see Supplementary Notes 4 and 5 and Supplementary Table 4 for additional details).

## Supplementary information

Supplementary Information

## Data Availability

All the data supporting the findings in this study are available in the manuscript and in the Supplementary Information. Further data and methods are available from the corresponding authors upon request.

## Code availability

All the codes used for this study are available from the corresponding authors upon reasonable request.
