# Supplementary material for: Boundary-directed epitaxy of block copolymers
Source: Nat Commun. 2020 Aug 19;11:4151. doi: 10.1038/s41467-020-17938-3 (PMC7438520; doi:10.1038/s41467-020-17938-3)
Supplement: Supplementary file 1 — Supplementary Information [file 41467_2020_17938_MOESM1_ESM.pdf]

Supplementary Information for:
Boundary-directed epitaxy of block copolymers

Jacobberger et al.

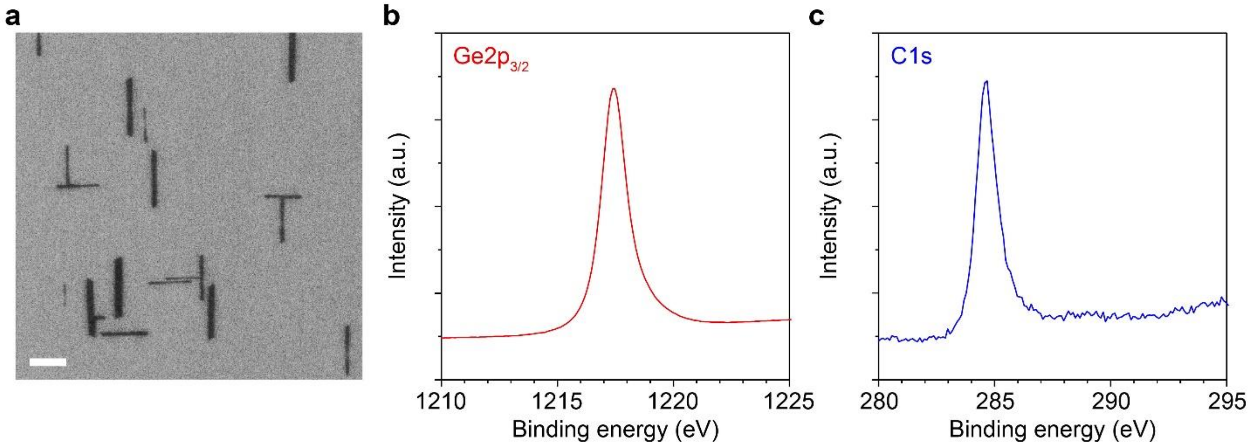

Supplementary Fig. 1 Characterization of unseeded graphene stripes on Ge(001). a, SEM image of a chemical pattern consisting of graphene stripes (darker contrast) grown on Ge(001) (lighter contrast). Stripes are roughly aligned along perpendicular Ge⟨110⟩ surface directions and nucleate at random locations and at random times. Scale bar is 500 nm. **b**,**c**, Plot of the Ge2$p_{3/2}$ (**b**) and C1s (**c**) XPS spectra for graphene stripes grown on Ge(001). Samples are transferred from the CVD system to the XPS chamber through air. Peaks corresponding to oxidized Ge and oxidized graphene are below the detection limit of ~0.1%. The atomic composition of all elements other than C and Ge is also below the detection limit of ~0.1%. These data indicate that before spin-coating BCP films for assembly studies, the graphene and Ge surfaces are relatively pristine and free of oxide and contaminants.

Supplementary Table 1 Properties of BCPs. Identity, domain spacing (L_0), molecular weight of PS (M_{PS}), molecular weight of PPC (M_{PPC}), polydispersity index (PDI), fraction of PPC by weight ($f_{PPC,wt}$), and fraction of PPC by volume ($f_{PPC,vol}$).

Sample	L_0 (nm)	M_{PS} (kg mol^{-1})	M_{PPC} (kg mol^{-1})	PDI	$f_{PPC,wt}$	$f_{PPC,vol}$
**PS-b-PPC**	16.8	7.6	8.0	1.01	0.51	0.47
**PPC-b-PS-b-PPC**	14.5	10.7	9.8	1.01	0.48	0.44
**PPC-b-PS-b-PPC**	12.8	9.8	8.5	1.01	0.47	0.43

Supplementary Note 1 Preferentiality of PPC and PS towards Ge and graphene. The effect of film thickness on hole/island formation[1] is studied to determine the preferentiality of PPC and PS towards Ge and graphene. Ge(001) substrates are annealed at 910 °C in a flow of 200 sccm of Ar and 100 sccm of H_2 for 2.5 h to achieve a Ge surface that is similar to the Ge surface directly after growth of graphene stripes. Continuous graphene films are grown on Ge(110) at 910 °C in a flow of 200 sccm of Ar, 100 sccm of H_2, and 4.6 sccm of CH_4 for 12 h. Ge(110) is used because the surface is relatively flat after growth, whereas the Ge(001) surface below graphene is rougher and forms hill-and-valley facets[2, 3]. PPC-b-PS-b-PPC (L_0 of 12.8 nm) is spin-coated onto bare Ge and continuous graphene surfaces at thicknesses of 1.0, 1.4, and 1.6L_0 and 1.1, 1.3, and 1.6L_0, respectively (Supplementary Fig. 2). The samples are then annealed at 160 °C for 15 min, and the resulting BCP film morphology is characterized with AFM (Supplementary Fig. 3–5).

In each case, the PPC-b-PS-b-PPC forms holes and islands with discrete thicknesses. The step height between holes and islands (Supplementary Fig. 3) as well as the percentage of the surface that they occupy (Supplementary Fig. 5) are determined (Supplementary Table 2). Next, H_2O contact angle measurements (Supplementary Fig. 6) and angle-resolved XPS studies (Fig. 4h and Supplementary Fig. 7) are conducted to determine whether PS or PPC wets the top, free surface of the holes and islands formed in Supplementary Fig. 3–4. These data indicate that PS preferentially wets the free surface, which is consistent with the lower surface energy of PS[4-6] compared to PPC[7].

After (1) the initial BCP film thickness after spin-coating (Supplementary Fig. 2), (2) the step height between holes and islands after annealing (Supplementary Fig. 3), (3) the fractional coverage of the surface by holes and islands after annealing (Supplementary Fig. 5), and (4) the identity of the polymer block that wets the free surface after annealing (Fig. 4f–h, Supplementary Fig. 6–7) are characterized, the structure of each film (Supplementary Fig. 8 and Supplementary Table 2) and the surface affinity of PPC and PS towards Ge and graphene are determined. These data indicate Ge and graphene are preferential to PPC and PS, respectively.

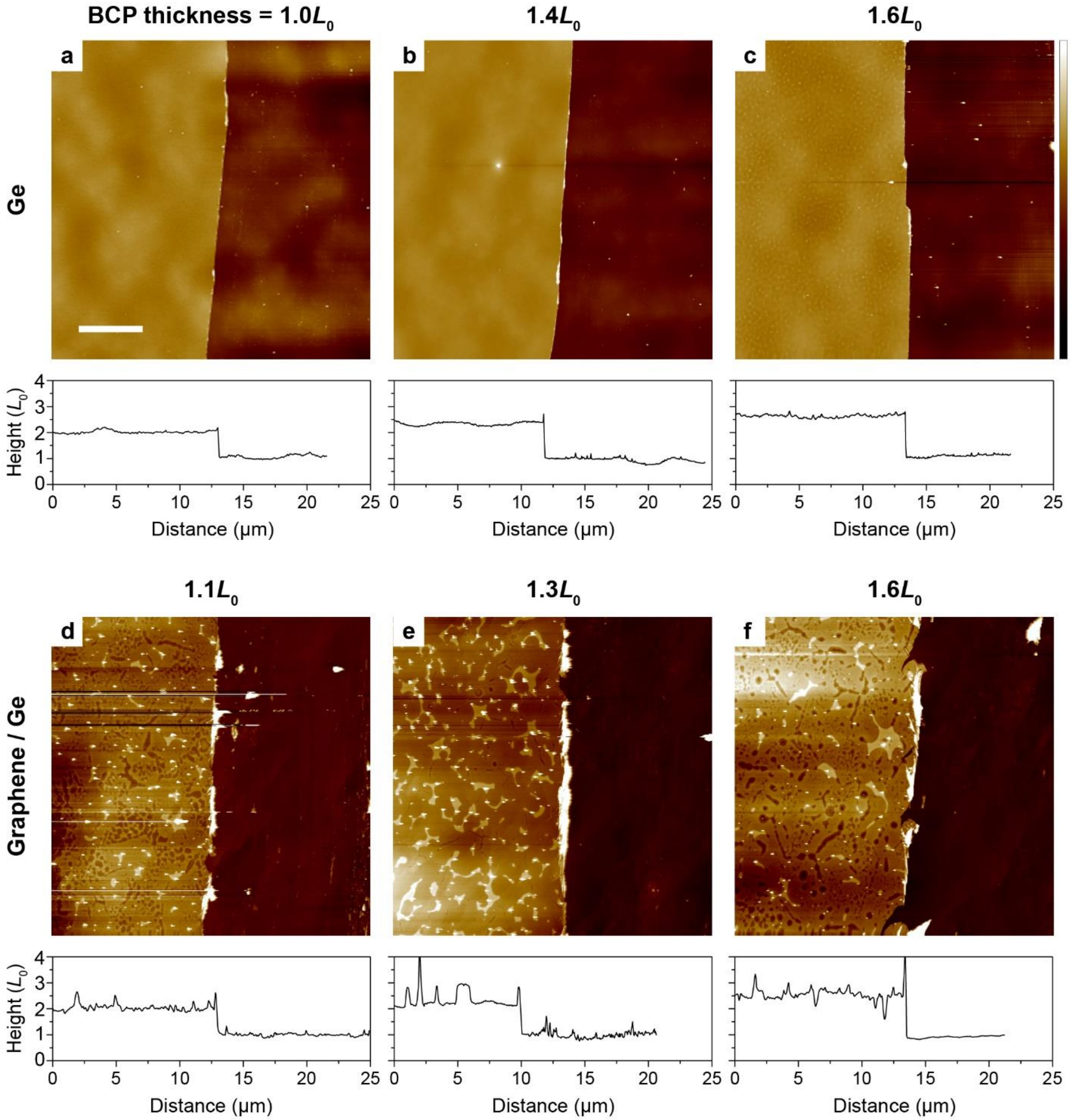

Supplementary Fig. 2 BCP film thickness after spin-coating. a-f, AFM topographic maps of PPC-b-PS-b-PPC (L_0 of 12.8 nm) films after spin-coating on bare Ge surfaces (**a-c**) and on continuous graphene films (**d-f**). Height profiles below each image indicate the corresponding film thicknesses, which are summarized in Supplementary Table 2. Scale bar is 5 μm and height scale is 60 nm.

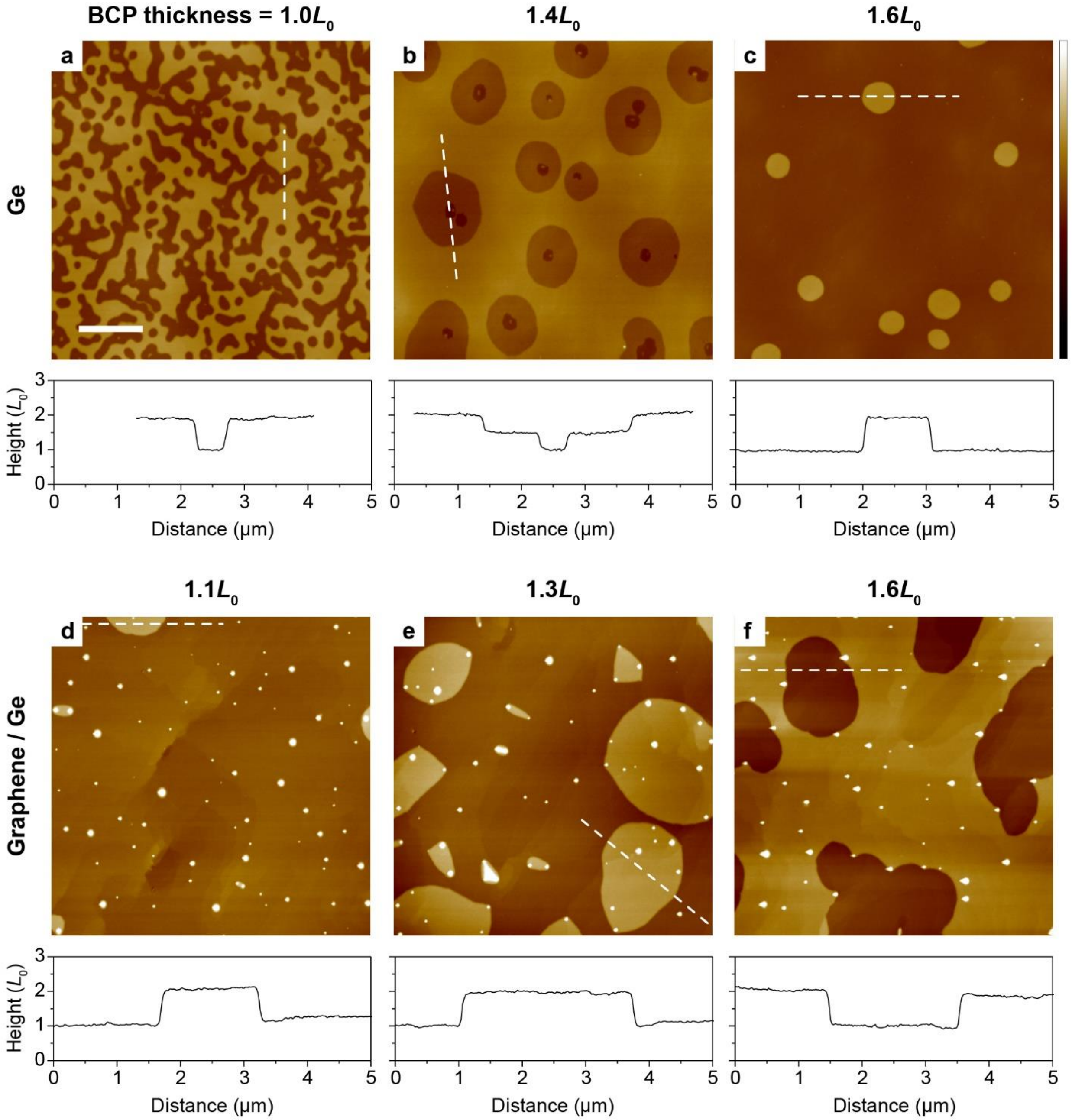

Supplementary Fig. 3 BCP topography after thermal annealing. a-f, AFM topographic maps of PPC-b-PS-b-PPC (L_0 of 12.8 nm) films on bare Ge surfaces (**a-c**) and on continuous graphene films (**d-f**) after annealing at 160 °C for 15 min. Height profiles below each image indicate the step height between regions of different thicknesses. Film thicknesses before annealing are 1.0 (**a**), 1.4 (**b**), 1.6 (**c**), 1.1 (**d**), 1.3 (**e**), and 1.6L_0 (**f**), as determined in Supplementary Fig. 2. Scale bar is 2 μm and height scale is 60 nm.

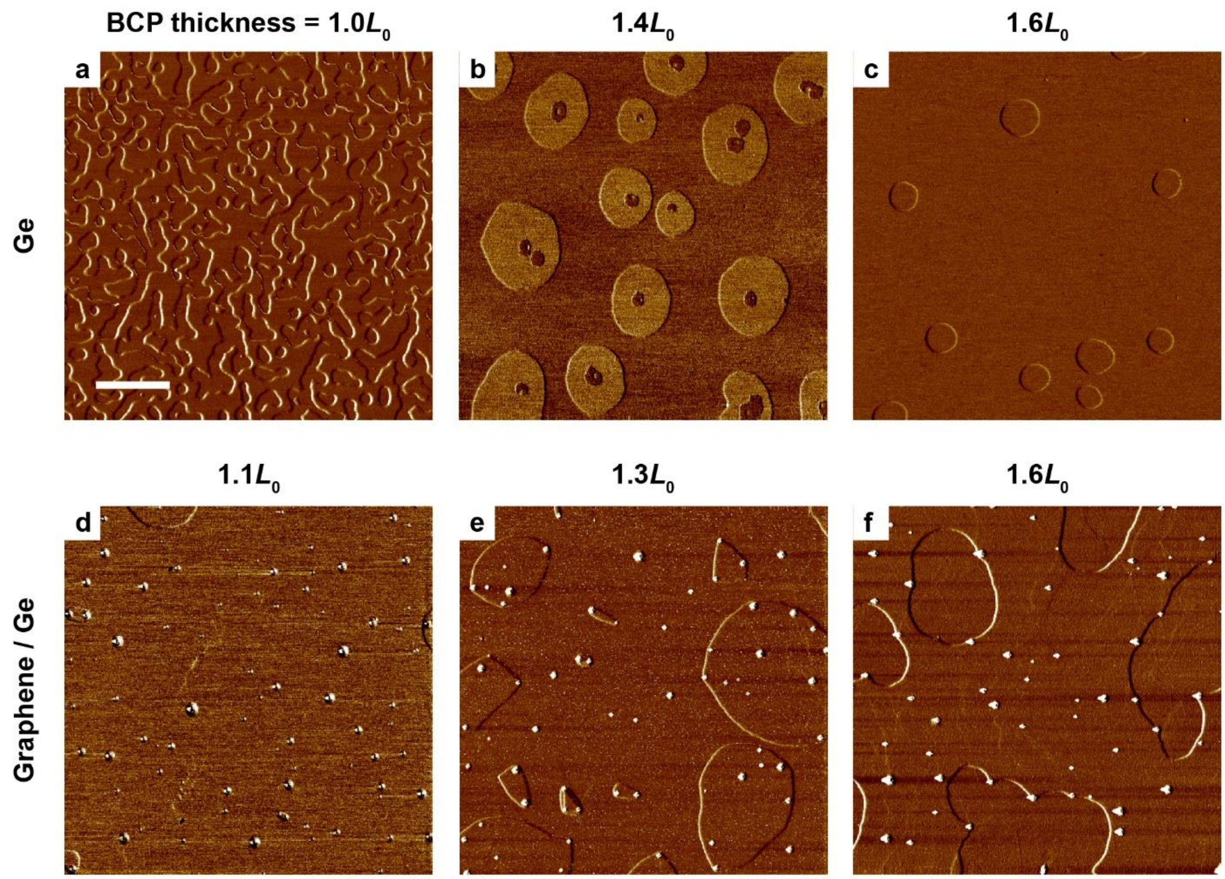

Supplementary Fig. 4 AFM phase images of BCP films after thermal annealing. a-f, AFM phase images of PPC-b-PS-b-PPC (L_0 of 12.8 nm) films on bare Ge surfaces (**a**-**c**) and on continuous graphene films (**d**-**f**) after annealing at 160 °C for 15 min, which correspond to the AFM height images in Supplementary Fig. 3. Scale bar is 2 μm.

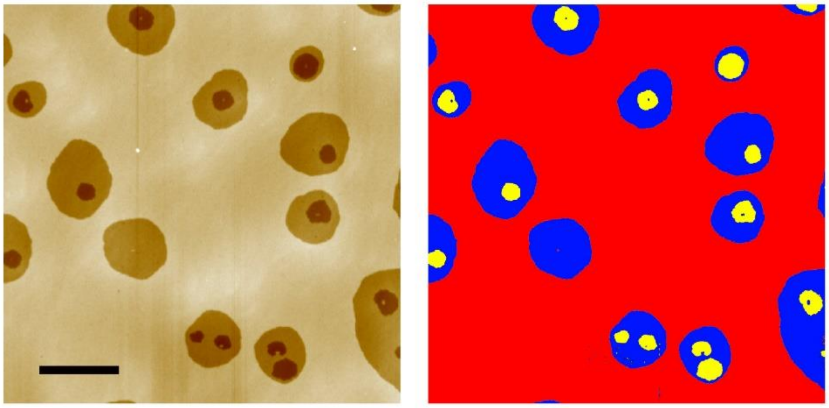

Supplementary Fig. 5 Quantification of hole and island coverage. AFM topographic map of PPC-b-PS-b-PPC (L_0 of 12.8 nm) after annealing at 160 °C for 15 min (left) shows three unique BCP heights (see Supplementary Fig. 3 for corresponding height profiles). Corresponding image (right) in which the red, blue, and yellow regions have film thicknesses of 2, 1.5, and 1L_0, respectively. From such images, the areal coverage of regions with each film thickness is determined. Scale bar is 2 μm.

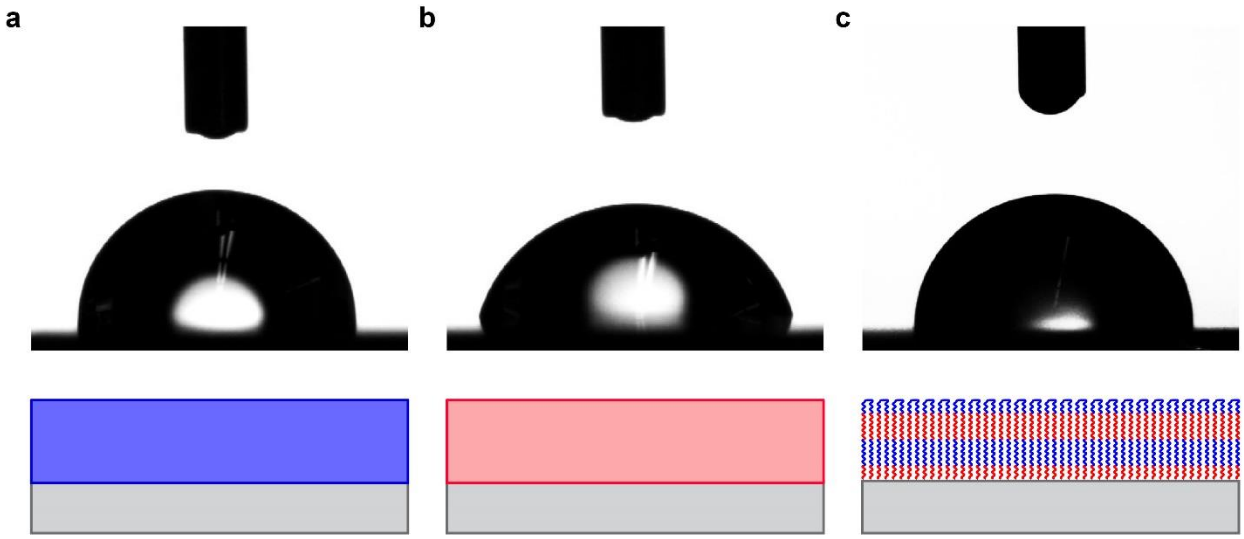

Supplementary Fig. 6 Characterization of BCP film surface via H_2O contact angle measurements. a-c, H_2O contact angle measurements on a PS homopolymer film (**a**), PPC homopolymer film (**b**), and PPC-b-PS-b-PPC (L_0 of 12.8 nm) film with thickness of $1.5L_0$ on Ge annealed at 160 °C for 15 min (**c**). H_2O contact angles are 90, 73, and 89° in **a-c**, respectively. These data indicate that PS (rather than PPC) preferentially wets the free surface during formation of holes and islands in the BCP film (as shown schematically in Supplementary Fig. 8). Schematic diagrams are shown for each substrate, in which blue, red, and grey correspond to PS, PPC and Ge, respectively.

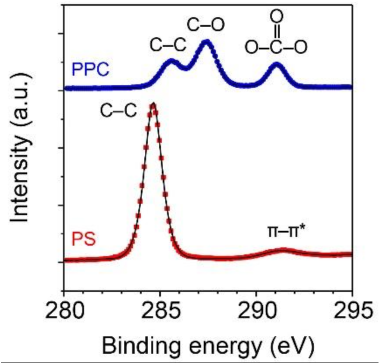

Supplementary Fig. 7 Characterization of PPC and PS films via XPS. Plot of the C1s XPS spectra for thick films of PS homopolymer (red squares) and PPC homopolymer (blue circles) with fits (black lines). The C─O and CO_3 peaks are only characteristic of PPC. Therefore, decreasing $I_{C\text{–}O}$ and I_{CO3} with respect to $I_{C\text{–}C}$ with increasing emission angle in Fig. 4h indicates that PS (rather than PPC) preferentially wets the free surface of holes and islands in the BCP film (as shown schematically in Supplementary Fig. 8).

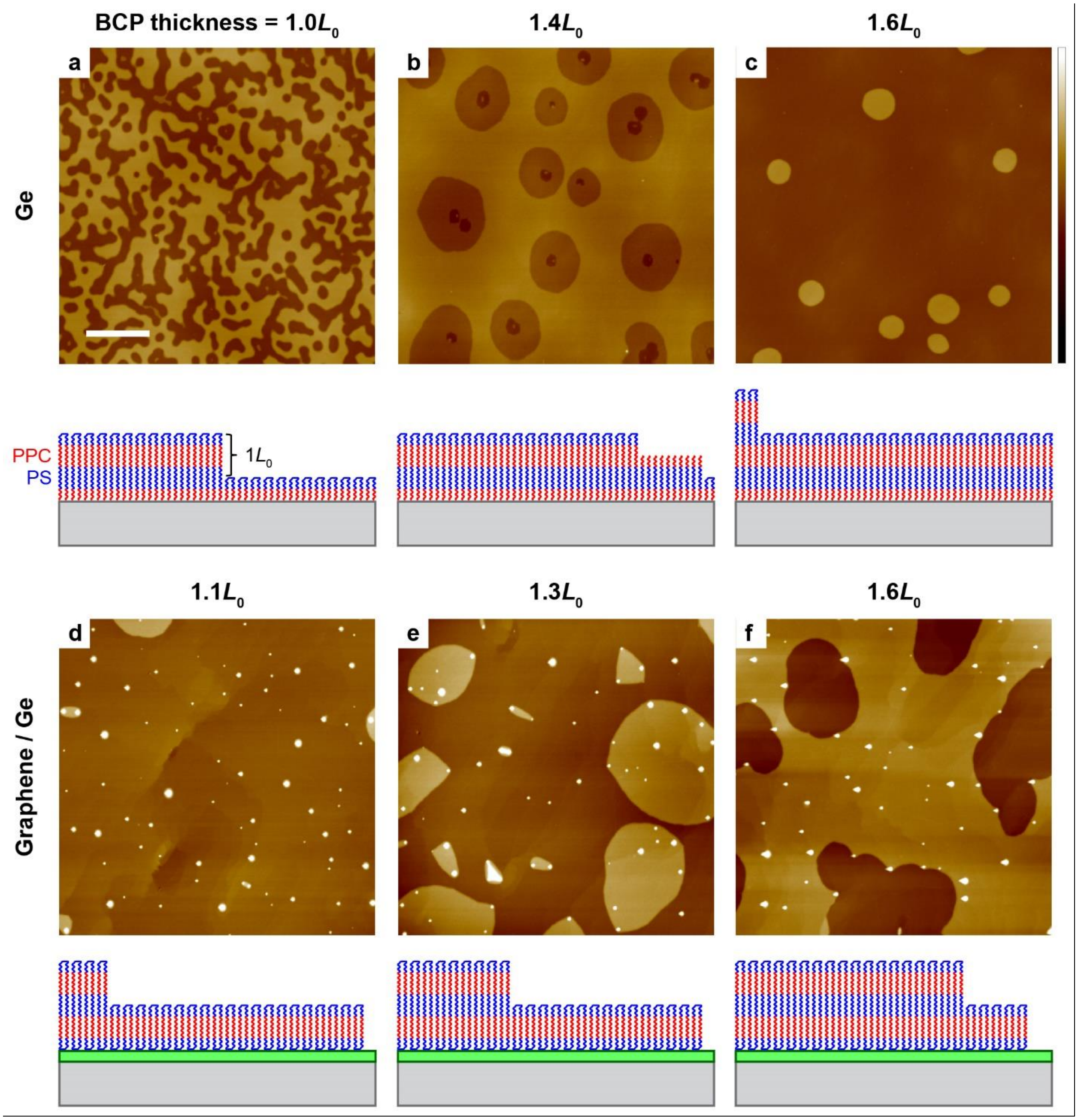

Supplementary Fig. 8 BCP topography determined from hole and island experiments. a-f, AFM topographic maps of PPC-b-PS-b-PPC (L_0 of 12.8 nm) films on bare Ge surfaces (**a-c**) and on continuous graphene films (**d-f**) after annealing at 160 °C for 15 min. Film thicknesses before annealing are 1.0 (**a**), 1.4 (**b**), 1.6 (**c**), 1.1 (**d**), 1.3 (**e**), and 1.6L_0 (**f**), as determined in Supplementary Fig. 2. Schematic diagrams show the corresponding side-view BCP film morphology, in which PS, PPC, Ge, and graphene are blue, red, grey, and green, respectively. The graphene thickness is exaggerated so that the graphene films can be visualized more easily. Scale bar is 2 μm and height scale is 60 nm. See Supplementary Note 1 for more information regarding the hole and island experiments.

Supplementary Table 2 Summary of the hole and island test. Thickness of PPC-b-PS-b-PPC (L_0 of 12.8 nm) films on Ge surfaces and on continuous graphene films measured directly via AFM after spin-coating (Supplementary Fig. 2) and determined from hole and island experiments (Supplementary Fig. 3–8).

Surface	Film thickness measured via AFM after spin-coating		Film thickness determined from hole and island experiment	
	Average (L_0)	Standard deviation (L_0)	Average (L_0)	Standard deviation (L_0)
**Ge**	1.62	0.08	1.57	0.06
	1.36	0.08	1.38	0.04
	0.98	0.08	1.01	0.05
**Graphene / Ge**	1.63	0.11	1.67	0.13
	1.26	0.11	1.33	0.06
	1.05	0.10	1.10	0.04

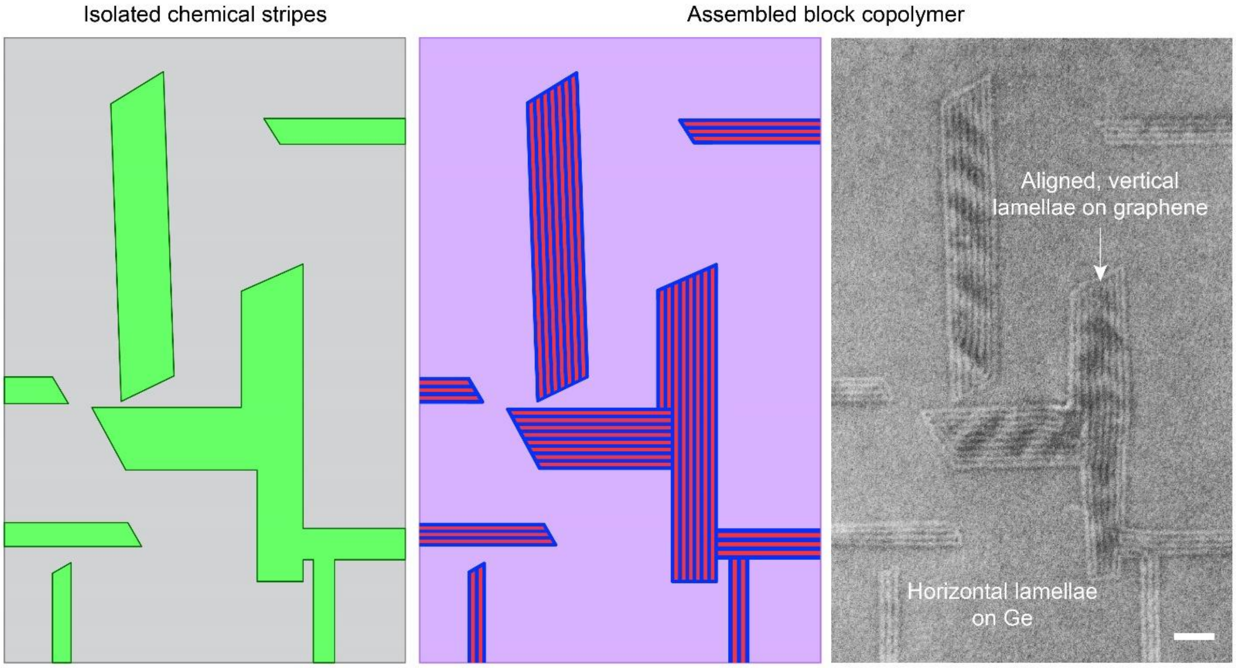

Supplementary Fig. 9 Boundary-directed epitaxy on unseeded graphene stripes. Schematic diagram of a chemical pattern consisting of polydisperse graphene stripes grown on a Ge(001) substrate without seeds (left)[3]. Schematic diagram (middle row) and SEM image (right) of PPC-b-PS (L_0 of 16.8 nm) after assembly. Graphene, Ge, vertical PS lamellae, vertical PPC lamellae, and horizontal lamellae are green, grey, blue, red, and purple, respectively. Scale bar is 100 nm.

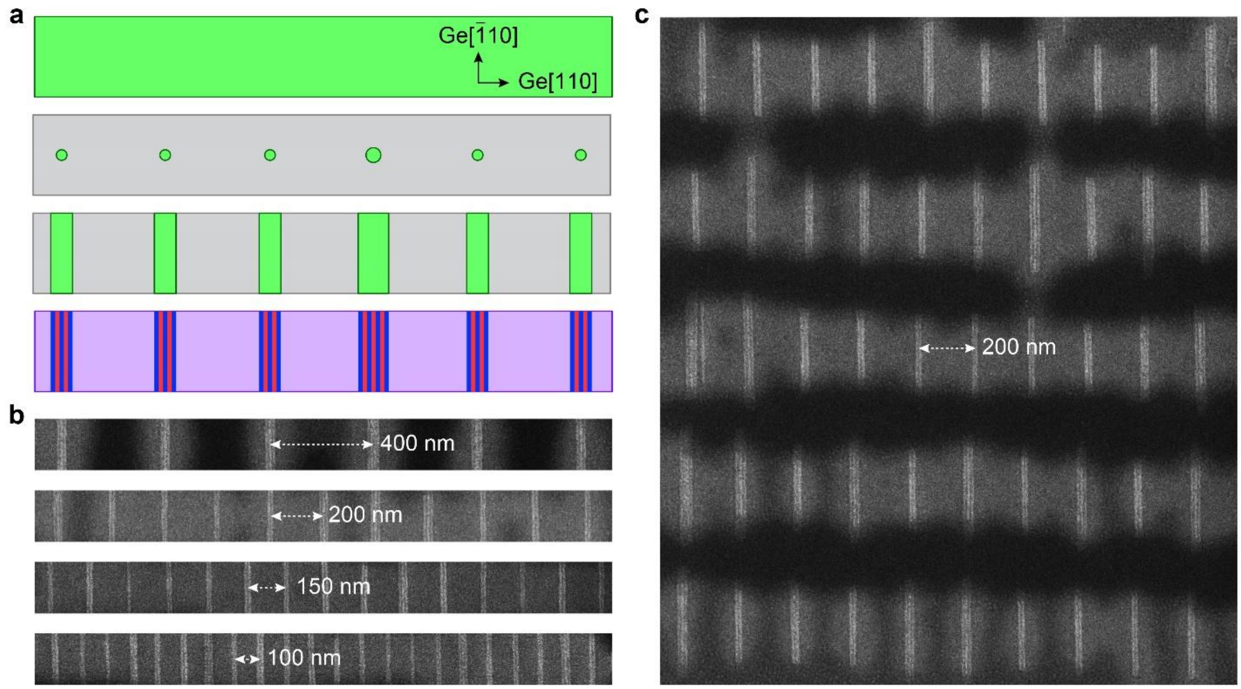

Supplementary Fig. 10 Boundary-directed epitaxy of line-array superstructures on seeded stripes. a, Schematic diagram after transfer of graphene onto Ge(001) with controlled crystallographic alignment, lithographic patterning of graphene into circular discs via electron-beam lithography and reactive ion etching, selective and anisotropic stripe growth from seed edges along a single Ge$\langle 110 \rangle$ direction[8, 9], and BCP assembly on the chemical pattern (top to bottom). The guiding scheme corresponds to the chemical pattern with pitch of 400 nm in **b**. Graphene, Ge, vertical PS lamellae, vertical PPC lamellae, and horizontal lamellae are green, grey, blue, red, and purple, respectively. **b**,**c**, SEM images of PPC-b-PS-b-PPC (L_0 of 12.8 nm) assembled on seeded graphene stripe arrays with varying superstructure pitch of 400, 200, 150, and 100 nm (top to bottom) (**b**) and with superstructure pitch in the horizontal and the vertical directions of 200 and 500 nm, respectively (**c**). Compared to assembly on unseeded chemical patterns (Supplementary Fig. 9), placement and alignment of BCP lamellae are more rationally controlled on seeded stripe arrays. Stripe-width polydispersity is reduced but not completely eliminated using seeds, and thus the number of vertical lamellae still varies from stripe to stripe.

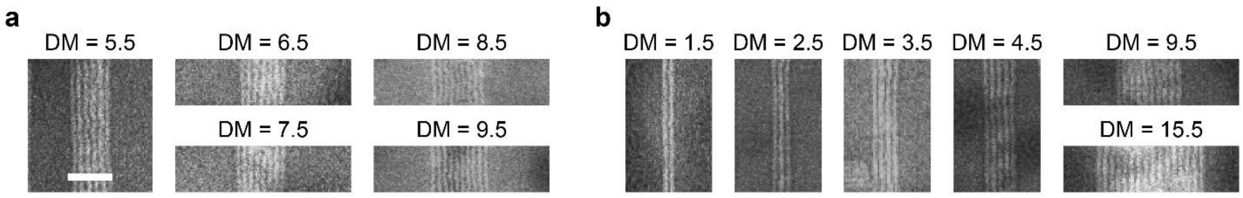

Supplementary Fig. 11 Density multiplication via boundary-directed epitaxy. a,**b**, SEM images of PPC-b-PS-b-PPC with L_0 of 12.8 (**a**) and 14.5 nm (**b**) assembled with various density multiplication factors (DM). Scale bar is 100 nm.

Anneal temperature (°C)	Anneal time (min)	Largest density multiplication factor
115	1.00E+01	1.5
115	1.32E+03	1.5
130	1.67E-02	1.5
130	2.50E-01	1.5
130	1.00E+01	1.5
130	6.00E+01	1.5
130	1.20E+02	1.5
160	8.33E-02	1.5
160	1.00E+00	1.5
190	8.33E-02	1.5
130	4.80E+02	2.5
145	1.00E+01	2.5
160	5.00E+00	2.5
175	1.00E+00	2.5
190	2.00E-01	2.5
130	7.20E+02	≥ 3.5
130	1.44E+03	≥ 3.5
130	5.04E+03	≥ 3.5
145	3.21E+03	≥ 3.5
160	1.00E+01	≥ 3.5
160	1.50E+01	≥ 3.5
160	2.00E+01	≥ 3.5
160	3.00E+01	≥ 3.5
175	2.50E+00	≥ 3.5
175	5.00E+00	≥ 3.5
175	1.00E+01	≥ 3.5
175	3.00E+01	≥ 3.5
190	5.00E-01	≥ 3.5
190	1.00E+00	≥ 3.5

Supplementary Table 3 Summary of assembly conditions. Largest density multiplication factor achieved after assembly with PPC-b-PS-b-PPC (L_0 of 12.8 nm) for various anneal temperatures and anneal times.

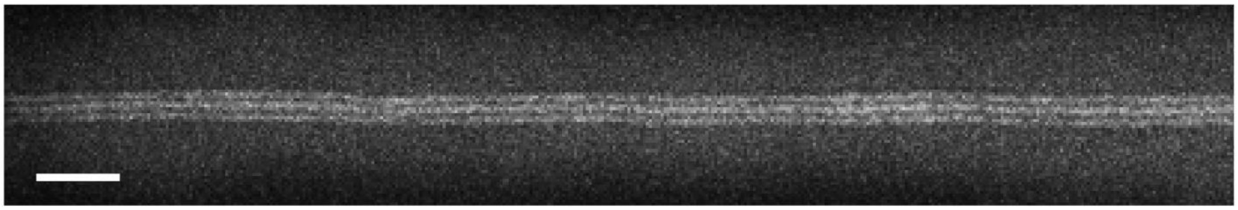

Supplementary Fig. 12 Density multiplication on a stripe with length of 1.5 µm. SEM image of PPC-b-PS-b-PPC (L_0 of 12.8 nm) assembled with a density multiplication factor of 2.5 on a stripe that is 1.5 µm in length. Scale bar is 100 nm.

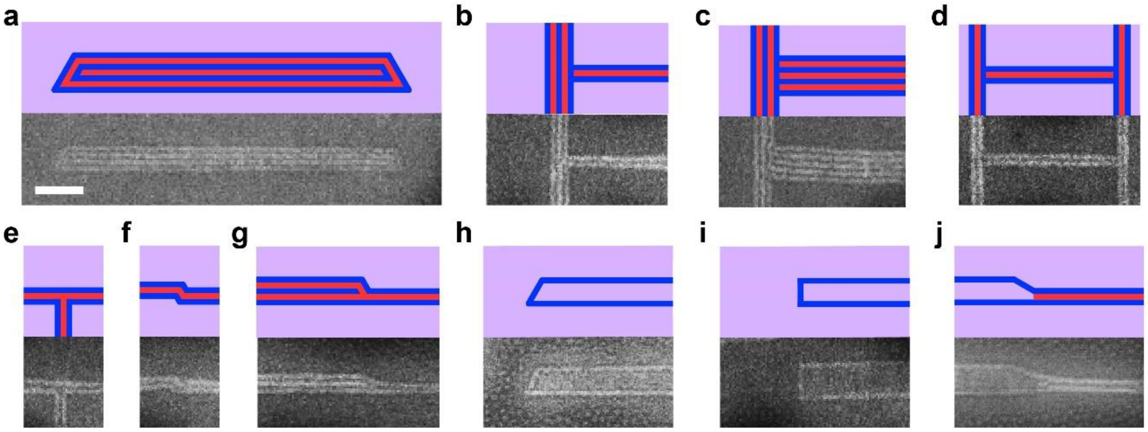

Supplementary Fig. 13 Assembly of isolated and complex features. a-j, PPC-b-PS-b-PPC (L_0 of 12.8 nm) assembled into various patterns, including an isolated line array with seven vertical lamellae (**a**), T-junctions with different numbers of vertical lamellae (**b-d**), three vertical lamellae splitting into two mirrored 90° bends, rather than forming T-junctions (**e**), a jog with three vertical lamellae (**f**), a jog similar to that in Fig. 2c except only the outermost PS line jogs when the stripe width changes (**g**), 60 and 120° bends with a single vertical lamella (**h**), 90° bends with a single vertical lamella (**i**), and two continuous vertical lamellae where their separation distance is varied, which is similar to the gate layer for NAND2 or NOR2 layout in integrated circuits[10]. Patterns in **h-j** are produced by partially assembling the BCP by annealing for a relatively short time so that only vertical PS lamellae at the stripe/substrate boundary are formed. Schematic diagrams (top) and SEM images (bottom) of the assembled BCP. Vertical PS and PPC lamellae are blue and red, respectively, and horizontal lamellae are purple. Scale bar is 100 nm.

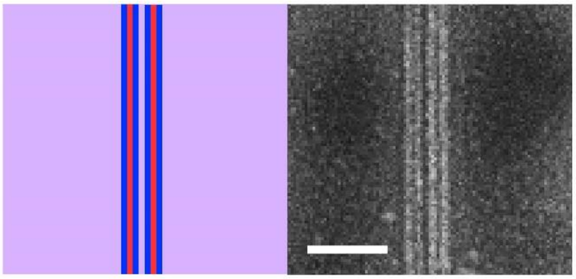

Supplementary Fig. 14 Boundary-directed epitaxy on tightly pitched stripes. Assembly of PPC-b-PS-b-PPC (L_0 of 12.8 nm) on stripes separated by a distance of roughly $0.5L_0$. Schematic diagram (left) and SEM images (right) of the assembled BCP. Vertical PS and PPC lamellae are blue and red, respectively, and horizontal lamellae are purple. Scale bar is 100 nm.

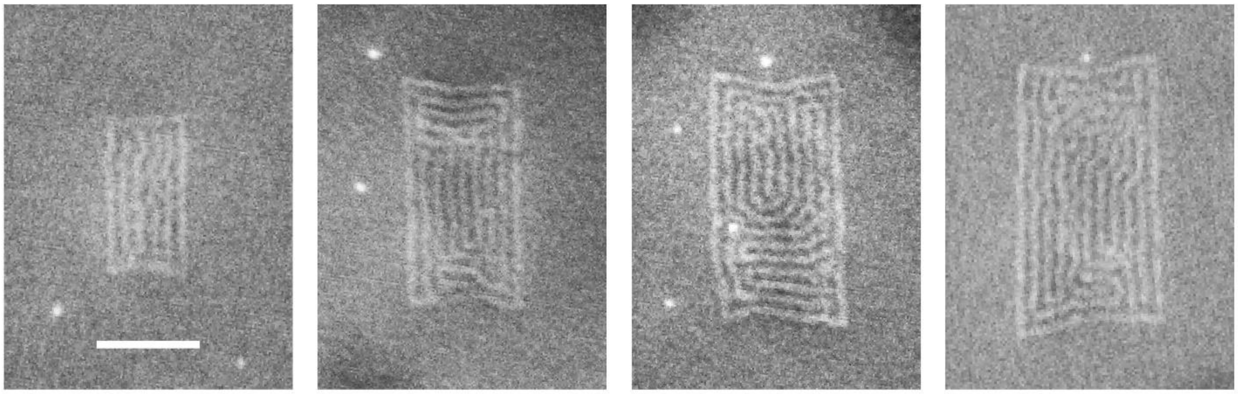

Supplementary Fig. 15 Boundary-directed epitaxy on stripes with different crystallographic edge orientations. Assembly of PPC-b-PS-b-PPC (L_0 of 12.8 nm) on isolated graphene stripes with edges that are not aligned along the armchair crystallographic direction of graphene. These stripes also have lower aspect ratios than stripes with armchair edges. On stripes with relatively low-aspect ratios, vertical lamellae can self-align to the stripe/substrate boundary along both the short edges and the long edges of the chemical stripes. Scale bar is 100 nm.

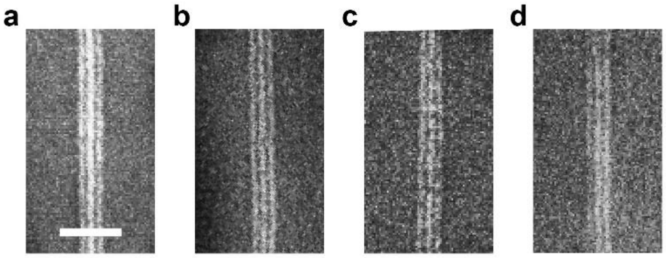

Supplementary Fig. 16 Boundary-directed epitaxy on various Ge surface orientations. a-d, Assembly of PPC-b-PS-b-PPC (L_0 of 12.8 nm) with density multiplication of 2.5 on graphene stripes on Ge(001) (**a**), Ge(110) (**b**), Ge(001) with 6° miscut towards Ge[110] (**c**), and Ge(001) with 9° miscut towards Ge[110] (**d**). Scale bar is 100 nm.

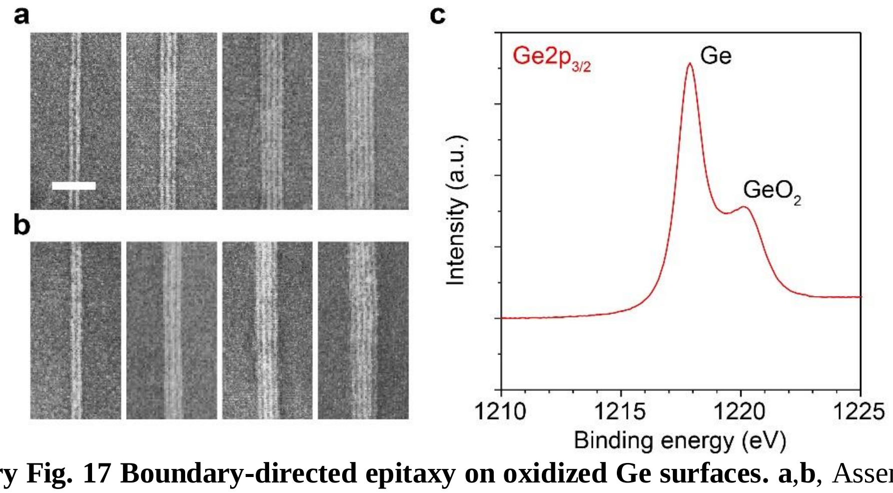

Supplementary Fig. 17 Boundary-directed epitaxy on oxidized Ge surfaces. a,**b**, Assembly of PPC-b-PS-b-PPC (L_0 of 12.8 nm) with density multiplication of 1.5, 2.5, 3.5, and 4.5 (left to right) on graphene stripes on unoxidized Ge as shown in Supplementary Fig. 1 (**a**) and oxidized Ge (**b**). Scale bar is 100 nm. **c**, Plot of the $Ge2p_{3/2}$ XPS spectrum for graphene stripes grown on oxidized Ge(001), as indicated by the presence of a substantial GeO_2 peak.

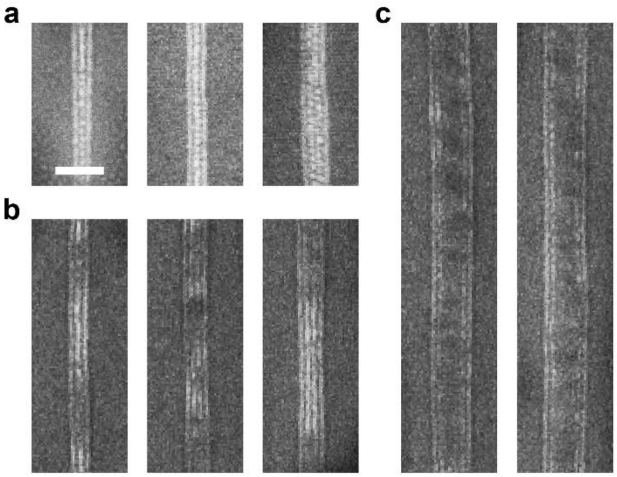

Supplementary Fig. 18 Incomplete assembly on graphene stripes. a-c, SEM images of PPC-b-PS-b-PPC (L_0 of 12.8 nm) after partial/incomplete assembly, in which vertical lamellae do not completely extend across the stripe width. **a**, Discontinuous dot-like domains on stripe widths of 2.5, 2.5, and 3.5L_0 (left to right). **b**, Simultaneous formation of vertical lamellae extending across the stripe width and horizontal lamellae on stripe widths of 2.5, 3.5, and 3.5L_0 (left to right). **c**, Partial formation of a second PS stripe adjacent to the continuous vertical PS lamella at both the left and right stripe edges. These data indicate that discontinuous dot-like domains (**a**) precede formation of continuous vertical lamellae in the stripe interior, as also observed in Fig. 3a. Furthermore, density multiplication is sometimes achieved at different locations along the stripe length at different times (**b**). We hypothesize that vertical lamellae preferentially assemble from the ends of existing isolated vertical lamellae. Finally, formation of vertical lamellae occurs directly bordering the continuous vertical PS lamellae that are pinned at both stripe edges. Scale bar is 100 nm.

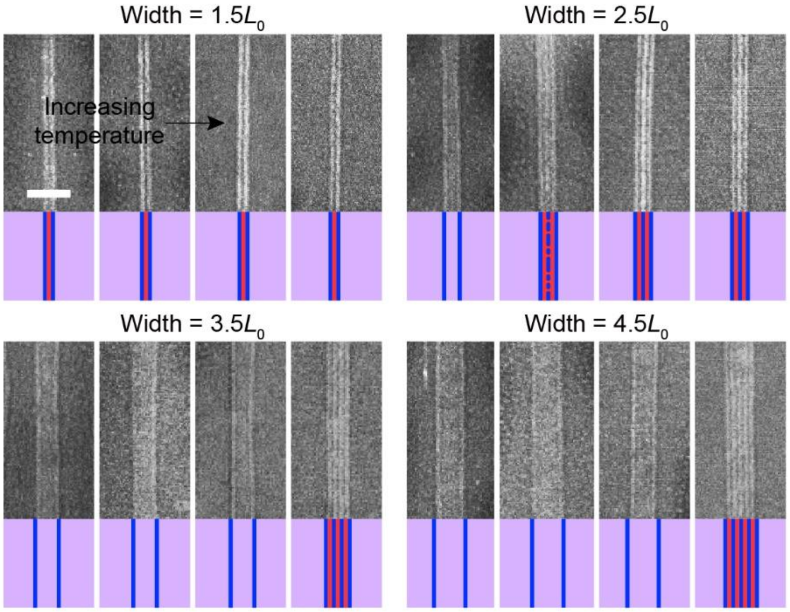

Supplementary Fig. 19 Effect of anneal temperature and stripe width on boundary-directed epitaxy. SEM images and schematic diagrams corresponding to PPC-b-PS-b-PPC (L_0 of 12.8 nm) morphology after assembly at 115, 130, 145, and 160 °C (left to right) for 10 min. Vertical PS and PPC lamellae are blue and red, respectively, and horizontal lamellae are purple. Scale bar is 100 nm.

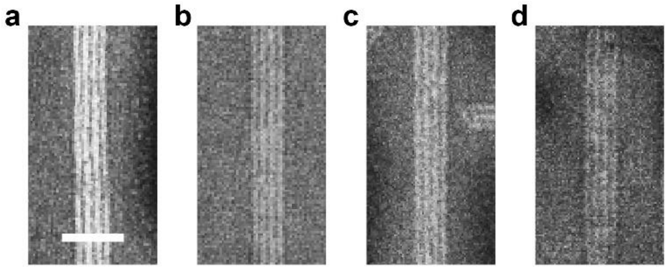

Supplementary Fig. 20 Effect of anneal temperature on assembly kinetics. a-d, Density multiplication of 3.5 is achieved using PPC-b-PS-b-PPC (L_0 of 12.8 nm) after annealing for roughly 24 h at 130 °C (**a**), 10 min at 160 °C (**b**), 2.5 min at 175 °C (**c**), and 30 s at 190 °C (**d**). Scale bar is 100 nm.

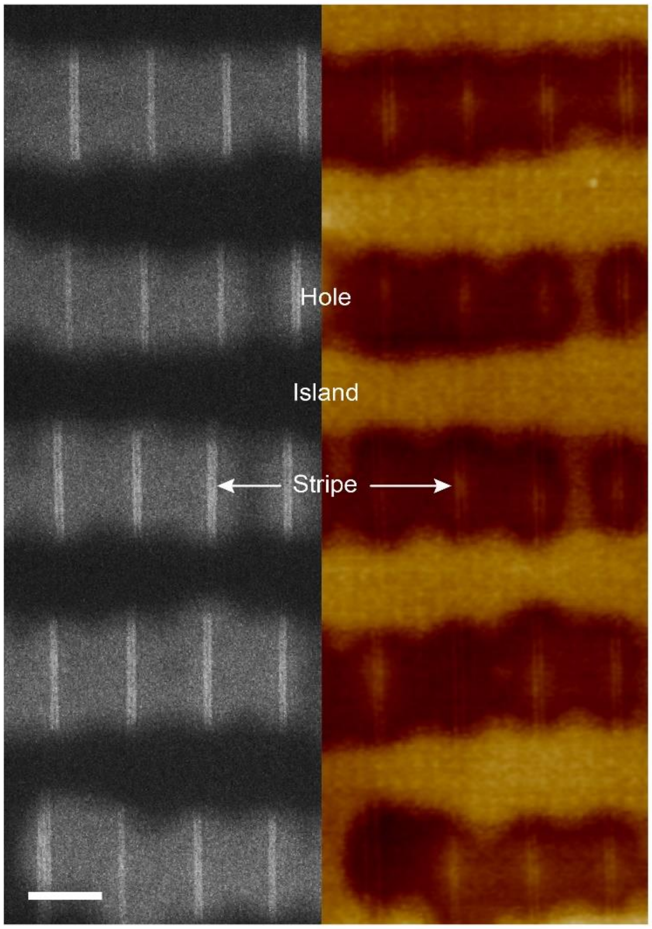

Supplementary Fig. 21 Hole and island formation on a stripe array. SEM (left) and corresponding AFM height (right) images of PPC-b-PS-b-PPC (L_0 of 12.8 nm) after assembly, in which the lighter and darker SEM contrast correspond to holes and islands, respectively, with step height of ~L_0 (Supplementary Fig. 21). Scale bar is 200 nm.

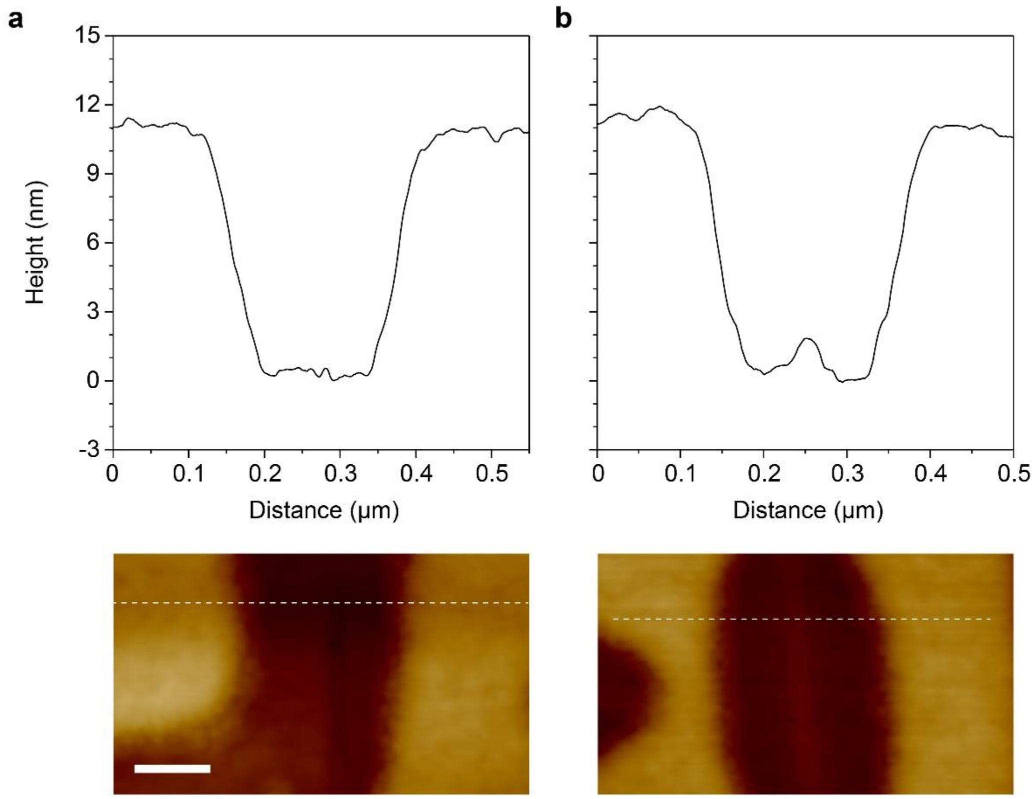

Supplementary Fig. 22 Hole/island step height on graphene/Ge chemical patterns. a,**b**, AFM profiles and AFM height images showing the step height between holes (in which graphene stripes are located) and islands in a PPC-b-PS-b-PPC (L_0 of 12.8 nm) film. Average step height is 10.5 nm, or 0.82L_0. White dashed lines in height images indicate the location at which profiles are measured. Scale bar is 100 nm.

Supplementary Note 2 Generalized theoretically informed coarse-grained model. In the main text, Monte Carlo (MC) simulations are conducted to further understand the assembly mechanism and elucidate the assembly evolution achieved experimentally in Fig. 3–4. To describe the system, a particle-based, theoretically informed coarse-grained (TICG) model with a generalized energy functional is used, in which each polymer chain is represented by a total of N coarse-grained beads.

The generalized TICG model used in this work describes the energetics of the following processes: (i) bonding between polymer beads, (ii) chemical incompatibility between unlike polymer beads, (iii) deviation of the local bead densities from their average value in a nearly incompressible, dense polymer melt, and (iv) interaction between surfaces in the system (i.e., the substrate, stripe, and free surface) and polymer beads. These interactions are parameterized by the following quantities: (i) the mean-squared end-to-end distance of an isolated, non-interacting chain, R_e, (ii) the incompatibility between beads, χN, (iii) the incompressibility, κN, and (iv) the interaction strength, λN.

Similar TICG models have been used extensively to understand the thermodynamics and kinetics of BCP assembly phenomena observed experimentally[11-13]. Even though TICG models do not precisely map to chemistry based on ab initio quantum theory, they have been validated by their ability to describe collective phenomena, such as phase separations, in multicomponent polymeric systems, which occur through the motion of macromolecules at length and time scales that are larger than more fundamental atomistic processes. Note, ab initio atomistic simulations are not a viable approach to model large, complex systems, such as those of interest in BCP assembly, due to limitations in computational resources.

In the generalized TICG model used here, the system consists of n A-b-B-b-A (representing PPC-b-PS-b-PPC) discretized chains with N = 32 coarse-grained beads per chain, in which the beads adapt a

Gaussian random-walk configuration. Bonded interactions within each polymer chain are described by harmonic springs attaching adjacent beads. The total harmonic potential of the system is defined as

$$H_b = \frac{3k_BT}{2}\frac{N-1}{Re^2}\sum_{k=1}^{n}\sum_{i=1}^{N-1}\boldsymbol{b}_k^2(i) \tag{1}$$

where $\boldsymbol{b}_k(i)$ is a vector connecting the ith and $(i+1)^{th}$ bead in a chain, k is the chain index, k_B is the Boltzmann constant, and T is the temperature. The non-bonded interactions are expressed as a functional of local densities of the A block, $\phi_A(\mathbf{r})$, and B block, $\phi_B(\mathbf{r})$[14]. The Hamiltonian contains both a quadratic term and a third-order term with respect to the local densities. Inclusion of the third-order term allows the coexistence of dense and sparse (e.g., vacuum) regions, which enables prediction of phenomena at the non-flat free surface at the top of the A-b-B-b-A film[15]. More specifically, the non-bonded interactions are given by

$$H_{nb} = \int_V \frac{d^3r}{Re^3}\left(\sum\sum\frac{v_{\alpha\beta}\phi_\alpha\phi_\beta}{2} + \sum\sum\sum\frac{\omega_{\alpha\beta\gamma}\phi_\alpha\phi_\beta\phi_\gamma}{3}\right) \tag{2}$$

where ϕ_α is the number density of polymer species, α, in a unit volume R_e^3, and integration is performed over the total volume, V, of the simulation box. The densities summation within the integral is performed over each species in the system (i.e., A and B). $v_{\alpha\beta}$ and $w_{\alpha\beta\gamma}$ are the second- and third-order virial coefficients, respectively, which describe the interaction between species, α, β, and γ, and affect the surface tension and average chain density of the BCP film. This energy functional has been used to understand phase behavior of a variety of systems, including BCPs[16], mixed polymer brushes[17, 18], and lipid bilayers[15].

For the polymer system containing two species (i.e., species A and B), the expanded form of the non-bonded interactions is defined as

$$H_{nb} = \int_V \frac{d^3r}{Re^3}\left(\begin{array}{c}\frac{v_{AA}\phi_A^2}{2} + \frac{v_{BB}\phi_B^2}{2} + v_{AB}\phi_A\phi_B + \\ \frac{w_{AAA}\phi_A^3}{3} + \frac{w_{BBB}\phi_B^3}{3} + w_{AAB}\phi_A^2\phi_B + w_{BBA}\phi_A\phi_B^2\end{array}\right) \tag{3}$$

Virial coefficients, v_{AA}, v_{BB}, w_{AAA}, and w_{BBB} are derived from the mean-field state equation of homopolymers[19] to have negligible vapor pressure and a finite coarse-grained compressibility of polymer melts. These terms are given by

$$v_{AA} = -2\frac{\kappa_A N + 3}{\sqrt{\bar{N}}} \tag{4}$$

$$v_{BB} = -2\frac{\kappa_B N + 3}{\sqrt{\bar{N}}} \tag{5}$$

$$w_{AAA} = \frac{3}{2}\frac{\kappa_A N + 2}{\sqrt{\bar{N}^2}} \tag{6}$$

$$w_{BBB} = \frac{3}{2}\frac{\kappa_B N + 2}{\sqrt{\bar{N}^2}} \tag{7}$$

where $\kappa_A N$ and $\kappa_B N$ are the isothermal incompressibility of A and B, respectively, and $\sqrt{\bar{N}}$ is the average chain density (i.e., the number of chains in a volume of R_e^3). As indicated by the sign of each parameter, v sets the attraction between beads, while w sets the repulsion between beads. The cross interaction parameter, v_{AB}, contains the Flory-Huggins parameter, χN and is defined as

$$v_{AB} = \frac{\chi N}{\sqrt{\bar{N}}} + \frac{v_{AA} + v_{BB}}{2} \tag{8}$$

The parameters w_{AAB} and w_{BBA} are expressed as arithmetic means of virial coefficients w_{AAA} and w_{BBB}, and are written explicitly as

$$w_{AAB} = \frac{2w_{AAA} + w_{BBB}}{3} \tag{9}$$

$$w_{BBA} = \frac{w_{AAA} + 2w_{BBB}}{3} \tag{10}$$

The average chain densities of A and B are set to 128. $\kappa_A N$ and $\kappa_B N$ are set to 200 and 100, respectively, reflecting the lower surface energy of PS compared to PPC. The order of magnitude of these values is guided by previously published results using similar TICG models[16, 20]. The value of χN is set to 35, which is slightly above the order-disorder transition point of A-b-B-b-A tri-BCPs[21].

Local densities of species within a particular region are calculated by splitting the simulation box into cubic grids, where the grid discretization length is $0.16R_e$. This technique, known as the particle-to-mesh (PM) method, is often used to simulate BCP assembly using TICG models in combination with MC simulations[14].

The surfaces of the substrate and stripe interact with the polymer beads following the potential

$$H_s = -\frac{\lambda}{d_s/R_e}\sum_i exp\left[-\frac{z_i^2}{2d_s^2}\right] \tag{11}$$

where z_i is the vertical distance of the ith bead from the bottom of the BCP film (i.e., at the stripe or substrate surface). The decay distance of the potential, d_s, is $0.15R_e$ and λN controls the interaction strength. The strength of the A–stripe, B–stripe, A–substrate, and B–substrate interactions are given by $\lambda_{\text{A-stripe}}N$, $\lambda_{\text{B-stripe}}N$, $\lambda_{\text{A-substrate}}N$, and $\lambda_{\text{B-substrate}}N$, respectively. The scale of these four λN values is chosen to be similar to values used in previous works that use a similar potential to model BCP assembly on patterned substrates[20, 22]. The relative values are adjusted to reproduce the experimental preferentiality of graphene to PS and Ge to PPC (Fig. 4f,g and Supplementary Fig. 2–8). In Fig. 5, $\lambda_{\text{A-stripe}}N$, $\lambda_{\text{B-stripe}}N$, $\lambda_{\text{A-substrate}}N$, and $\lambda_{\text{B-substrate}}N$ are -2.0, -2.35, -2.5 and -2.0, respectively. Additional simulations are conducted to elucidate the effect of chemical preferentially, BCP surface energy, BCP film thickness, step height at the stripe/substrate boundaries, and stripe width on assembly (see Supplementary Note 4,5 and Supplementary Fig. 23–27,29). The values for χN, $\kappa_B N$, $\kappa_A N$, $\lambda_{\text{B-stripe}}N$, $\lambda_{\text{A-stripe}}N$, $\lambda_{\text{B-substrate}}N$, $\lambda_{\text{A-substrate}}N$, number of MC cycles, and initial relaxed BCP film thickness for each of the simulations conducted in this work are provided in Supplementary Table 4.

Supplementary Note 3 Dynamic Monte Carlo simulations of boundary-directed epitaxy. Dynamic MC simulations are conducted to evolve the system and gain insight into the mechanisms governing boundary-directed epitaxy. Dynamic MC simulations have been previously used to understand the kinetics of self-assembly in polymeric systems and nanoparticle systems[23-25].

In this technique, a randomly selected bead is proposed to move in all three dimensions by a random distance in the range [$-dL$, dL], where dL is $0.8b$ and b is the mean-squared bond length of an ideal chain. Since trial moves consider only local displacement of randomly selected beads, translation or switching of entire chains is not possible. Therefore, collective motion occurring during the assembly evolution can be simulated, even if the detailed chain dynamics are not precisely captured. The proposed trial move is accepted with a probability of min(1, $e^{-\Delta H}$), where ΔH is the difference between the initial and final energies (i.e., the Metropolis criteria). One MC cycle contains a total of nN trial moves (where nN is the total number of beads in the system). The acceptance probability of trial moves obtained using this approach is ~0.35.

The dimensions of the simulation box in the x, y, and z dimensions (L_x, L_y, and L_z, respectively) are $17.5R_e$, $7.5R_e$, and $3.75R_e$, respectively, where L_0 is ~$1.25R_e$. Periodic boundary conditions are applied in the lateral x and y directions, while hard walls are placed at the bottom and top of the simulation box in the z-direction. A randomly distributed A-b-B-b-A BCP film with initial thickness of $1.25R_e$ and initial chain density of $128R_e^{-3}$ is placed on the stripe/substrate template at the bottom of the simulation box. The initial BCP film thickness is less than L_z, resulting in free, unfilled space above the BCP film. On the order of 10^6 dynamic MC cycles are performed to allow the system to approach equilibrium, enabling observation of the BCP assembly evolution during boundary-directed epitaxy.

Figure	Panel	χN	$\kappa_B N$	$\kappa_A N$	$\lambda_{B\text{-stripe}}N$	$\lambda_{A\text{-stripe}}N$	$\lambda_{B\text{-substrate}}N$	$\lambda_{A\text{-substrate}}N$	MC cycles	BCP film thickness (L_0)
5	a	35	100	200	-2.35	-2	-2	-2.5	1.00E+02	1.15
5	b	35	100	200	-2.35	-2	-2	-2.5	6.50E+04	1.15
5	c	35	100	200	-2.35	-2	-2	-2.5	1.25E+05	1.15
5	d	35	100	200	-2.35	-2	-2	-2.5	1.80E+05	1.15
5	e	35	100	200	-2.35	-2	-2	-2.5	3.00E+05	1.15
5	f	35	100	200	-2.35	-2	-2	-2.5	2.15E+06	1.15
S23	a	35	100	200	-2.35	-2	-2	-2.5	3.00E+06	1.15
S23	b	35	100	200	-2.35	-2	-2	-2.5	2.50E+06	1.15
S24	a	35	100	150	-2.35	-2	-2	-2.5	2.00E+06	1.15
S24	b	35	100	200	-2.35	-2	-2	-2.5	2.15E+06	1.15
S24	c	35	100	250	-2.35	-2	-2	-2.5	1.70E+06	1.15
S24	d	35	100	200	-2.75	-2	-2	-2.5	2.00E+06	1.15
S25	NA	35	100	200	-2	-2.5	-2.35	-2	1.55E+06	1.15
S26	a	35	100	200	-2.35	-2	-2	-2.5	2.00E+06	1.3
S26	b	35	100	200	-2.35	-2	-2	-2.5	2.00E+06	0.95
S26	c	35	100	200	-2.35	-2	-2	-2.5	5.00E+04	0.8
S26	d	35	100	200	-2.35	-2	-2	-2.5	2.00E+06	0.8
S27	NA	35	100	200	-2.35	-2	-2	-2.5	1.55E+06	1.15
S29	a	35	100	100	-2	-2.35	-2	-2	2.50E+05	1.3
S29	b	35	100	100	-2	-2.35	-2	-2	3.00E+06	1.3
S29	c	35	100	100	-2	-2	-2	-2.5	2.50E+05	1.3
S29	d	35	100	100	-2	-2	-2	-2.5	3.00E+06	1.3

Supplementary Table 4 Summary of simulation parameters. The values for χN, $\kappa_B N$, $\kappa_A N$, $\lambda_{B\text{-stripe}}N$, $\lambda_{A\text{-stripe}}N$, $\lambda_{B\text{-substrate}}N$, $\lambda_{A\text{-substrate}}N$, number of MC cycles, and initial relaxed BCP film thickness (after a few MC cycles) for each figure that shows MC simulation results are provided.

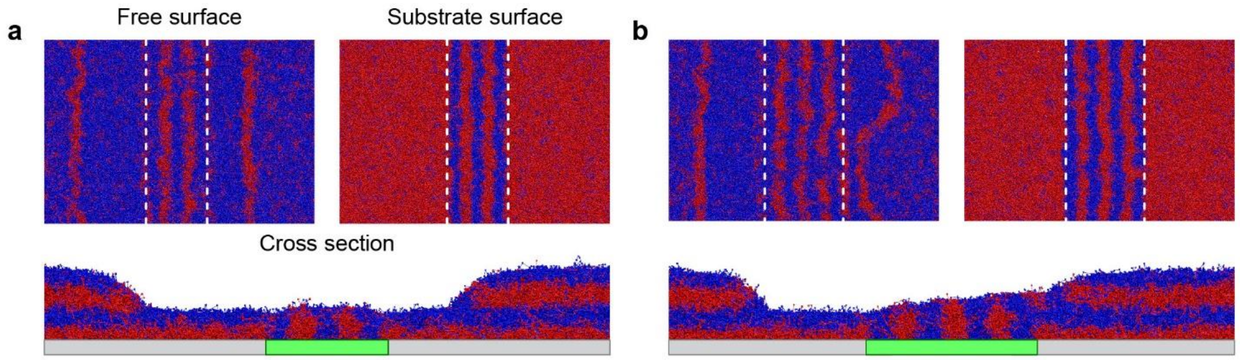

Supplementary Fig. 23 Monte Carlo simulations of boundary-directed epitaxy with density multiplication. a,**b**, Simulated BCP morphology at the free surface (top left), at the substrate surface (top right), and along a cross-section perpendicular to the stripe width (bottom) on a stripe with width of $2.5L_0$ (**a**) and $3.5L_0$ (**b**), corresponding to density multiplication factors of 2.5 and 3.5, respectively. In contrast, in Fig. 5, the stripe width is $1.5L_0$. The BCP morphology in **b** has not reached equilibrium, as the L-junction at the right stripe/substrate boundary has not completely formed. The stripe, substrate, B, and A are green, grey, blue, and red, respectively. White dashed lines indicate the positions of the stripe/substrate boundaries.

Supplementary Note 4 Assembly pathway in boundary-directed epitaxy. Next, the energetic factors that dictate the evolution from an initially disordered state (Fig. 5a) to a more relaxed ordered morphology observed after boundary-directed epitaxy (Fig. 5f)—in which vertical and horizontal lamellae form on the stripe and substrate, respectively, and L-junctions form at the stripe/substrate boundaries—are discussed.

The free energy of the system includes contributions from (i) the interaction of A and B with the substrate and stripe, (ii) the surface tension of A and B at the free surface, (iii) the interfacial interaction between A and B, and (iv) the conformation of the polymer chains. The preferentiality of the stripe to B (i.e., graphene to PS), the substrate to A (i.e., Ge to PPC), and the free surface to B (i.e., the free surface to PS) are established experimentally (see Fig. 4f-h and Supplementary Fig. 2–8), indicating that $\gamma_{\text{A-stripe}} > \gamma_{\text{B-stripe}}$, $\gamma_{\text{B-substrate}} > \gamma_{\text{A-substrate}}$, and $\gamma_{\text{A-free surface}} > \gamma_{\text{B-free surface}}$, where $\gamma_{\alpha\text{-}\beta}$ is the interfacial energy between α and β, or the surface tension of α when β is the free surface. Calculation of the free energies is beyond the scope of this work; instead, the energetic factors that drive the BCP to assemble via the pathway observed in Fig. 5 are discussed qualitatively.

Upon annealing the initially disordered A-b-B-b-A film (Fig. 5a), horizontal B-A-B and A-B-A lamellae form on the stripe and substrate, respectively, due to preferential wetting (Fig. 5b). At the stripe/substrate boundaries, a curved three-dimensional network connects B and A domains across the antiphase transition. The system evolves from Fig. 5a to Fig. 5b by increasing favorable contact between B and the stripe ($\gamma_{\text{A-stripe}} > \gamma_{\text{B-stripe}}$) and increasing favorable contact between A and the substrate ($\gamma_{\text{B-substrate}} > \gamma_{\text{A-substrate}}$).

Upon further annealing, a mass transfer event is initiated, in which the BCP partially flows off the stripe and onto the substrate, forming horizontal A-B-A-B lamellae on the substrate (Fig. 5c,d). The system evolves from Fig. 5b to Fig. 5c,d by increasing favorable contact between B and the free surface ($\gamma_{\text{A-free surface}} > \gamma_{\text{B-free surface}}$) above the substrate, and by reducing the height of the energetically unfavorable antiphase transitions at the stripe/substrate boundaries. However, the former is likely the primary driving force acting to reduce the thickness of the BCP film at the stripe/substrate boundaries. For example, when the incompressibility of A is reduced to become more similar to the incompressibility of B—effectively reducing the energy penalty of A in contact with the free surface—the antiphase transition in Fig. 5b is stabilized and the mass transfer event in Fig. 5c,d is not observed (Supplementary Fig. 24a). Thus, it is likely the energetic favorability of horizontal A-B-A-B (as opposed to A-B-A) lamellae on the substrate that primarily drives the BCP film to become thinner in the vicinity of the stripe.

As mass continues to transfer from the stripe to the substrate, slanted lamellae that curve upward and contact the free surface form above the substrate at the stripe/substrate boundaries (Fig. 5d,e). The

formation of slanted lamellae is to some extent driven by relieving energetically unfavorable strain in the polymer chains, as the BCP morphology near the stripe/substrate boundaries in Fig. 5b,c significantly deviates from a lamellar structure with periodicity of L_0. Similar slanted lamellae have been observed at step edges in di-BCP films[26] and at the curved surface of BCP droplets[20, 27]. These slanted lamellae subsequently drive the nucleation of vertical lamellae above the stripe at the stripe/substrate boundaries (Fig. 5e). With further annealing, the BCP continues to diffuse from the stripe to the substrate so that the horizontal lamellae on the substrate approach the commensurate thicknesses of $0.5L_0$ in holes and $1.5L_0$ in islands (Fig. 5f and Supplementary Fig. 24b). During this process, L-junctions form at the stripe/substrate boundaries, matching the BCP morphology observed experimentally (Fig. 1,3,4).

The discussion above highlights that a decrease in the BCP film thickness near the stripe/substrate boundaries—primarily driven by a reduction in unfavorable contact of A with the free surface—is an important step that leads to the formation of slanted lamellae and the subsequent nucleation of vertical lamellae on the stripe. However, even if thinning of the BCP at the stripe/substrate boundaries occurs, boundary-directed epitaxy is only realized if (1) the interaction between the stripe and B is not too strong and (2) the interaction between B and the free surface is not too strong. If (1) and (2) are not satisfied, vertical lamellae do not form because it is either (1) too energetically unfavorable for A to contact the free surface above the stripe, resulting in horizontal A-B lamellae on the stripe (Supplementary Fig. 24c), or (2) too energetically unfavorable for A to wet the surface of the stripe, resulting in horizontal B-A-B lamellae on the stripe (Supplementary Fig. 24d). The effect of chemical preferentiality and BCP surface energy on boundary-directed epitaxy are discussed further, below, in Supplementary Note 5.

Finally, it is also useful to consider why the BCP morphology in Fig. 5f does not continue to evolve so that vertical lamellae propagate from the stripe/substrate boundaries onto the substrate surface. If vertical lamellae propagate onto the substrate, an energy penalty would be incurred with each additional vertical lamella due to increased unfavorable contact of B with the substrate and increased unfavorable contact of A with the free surface. However, there would not be an energetic stabilization associated with the addition of each vertical lamella, as the rest of the system, including the L-junctions at the interface between horizontal and vertical lamellae, would essentially remain the same. Therefore, unlike formation of vertical lamellae on the stripe—which is favorable due to the narrowness of the stripe and the energetic penalty associated with the antiphase transitions at the stripe/substrate boundaries—formation of vertical lamellae on the substrate is unfavorable and does not occur.

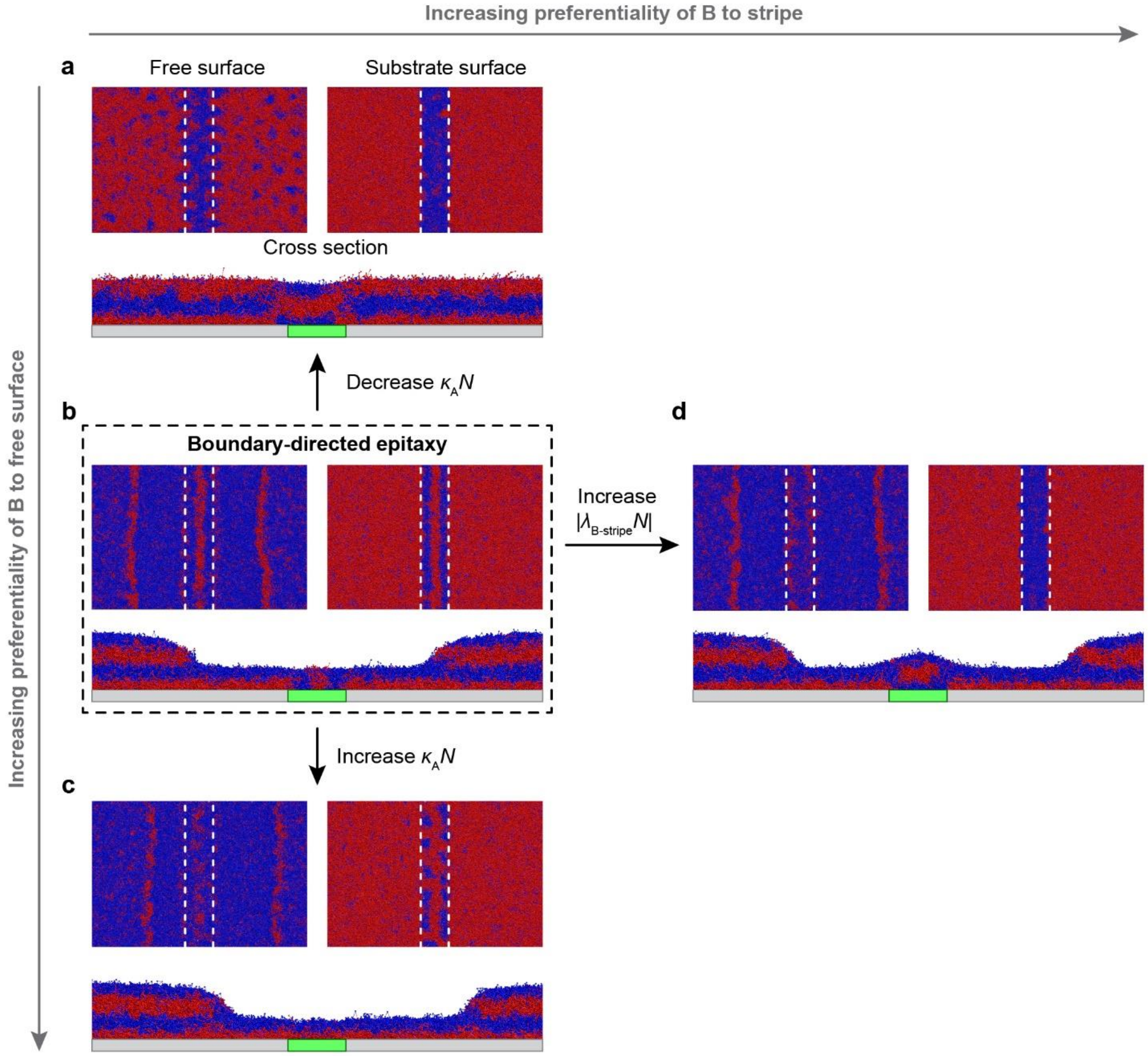

Supplementary Fig. 24 Monte Carlo simulations with varying preferentiality. a-d, Simulated BCP morphology at the free surface (top left), at the substrate surface (top right), and along a cross-section perpendicular to the stripe width (bottom) on a stripe with width of $1.5L_0$ for constant $\lambda_{\text{B-stripe}}N$, $\lambda_{\text{B-substrate}}N$, $\lambda_{\text{A-stripe}}N$, and $\lambda_{\text{A-substrate}}N$, and $\kappa_{\text{B}}N$ of -2.35, -2.0, -2.0, -2.5, and 100, respectively, and varying $\kappa_{\text{A}}N$ of 150 (**a**), 200 (**b**), and 250 (**b**), as well as for constant $\lambda_{\text{B-substrate}}N$, $\lambda_{\text{A-stripe}}N$, and $\lambda_{\text{A-substrate}}N$, $\kappa_{\text{B}}N$, and $\kappa_{\text{A}}N$ of -2.0, -2.0, -2.5, 100, and 200, respectively, and varying $\lambda_{\text{B-stripe}}N$ of -2.35 (**b**) and -2.75 (**d**). Consequently, $\kappa_{\text{A}}N$ increases from **a** to **b** to **c**, and $|\lambda_{\text{B-stripe}}N|$ increases from **b** to **d**. The stripe, substrate, B, and A are green, grey, blue, and red, respectively. White dashed lines indicate the positions of the stripe/substrate boundaries.

Supplementary Note 5 Monte Carlo simulations of assembly as a function of chemical preferentially, block copolymer incompressibility, and block copolymer film thickness.

Effect of chemical preferentiality on boundary-directed epitaxy. The window of chemical contrast required for boundary-directed epitaxy is elucidated. Monte Carlo simulations are conducted in which $\lambda_{\text{B-stripe}}N$ and $\lambda_{\text{A-substrate}}N$ are independently varied from -2.75 to -2.25 in steps of 0.05, while the stripe width, $\lambda_{\text{A-stripe}}N$, and $\lambda_{\text{B-substrate}}N$ are constant at $1.5L_0$, -2.0, and -2.0, respectively. Larger negative values of λ indicate a stronger attractive interaction. The MC simulations show that a sufficiently strong attraction between the substrate and A (i.e., $\lambda_{\text{A-substrate}}N < -2.45$) is required for boundary-directed epitaxy. Provided that $\lambda_{\text{A-substrate}}N < -2.45$, there exists a wide window of $\lambda_{\text{B-stripe}}N$ from -2.35 to -2.60 in which boundary-directed epitaxy is achieved (Supplementary Fig. 24b). When the attraction between the stripe and B is too weak (i.e., $\lambda_{\text{B-stripe}}N > -2.30$), both A and B wet the stripe to form vertical lamellae. However, vertical lamellae aligned perpendicular to the stripe/substrate boundaries (as observed in Shin et al.[28]) are stabilized over vertical lamellae aligned parallel to the stripe/substrate boundaries (as observed in boundary-directed epitaxy). In contrast, when the attraction between the stripe and B is too strong (i.e., $\lambda_{\text{B-stripe}}N < -2.65$), horizontal lamellae form on both the stripe and the substrate (Supplementary Fig. 24d) because it is too energetically unfavorable for A to wet the stripe to form vertical lamellae.

Boundary-directed epitaxy is not realized when the stripe and substrate materials are exchanged (Supplementary Fig. 25), representing a Ge stripe on a graphene substrate and reversing the chemical contrast scheme used in Fig. 5. Initially, horizontal lamellae form on both the stripe and substrate, with an antiphase transition at the stripe/substrate boundaries (like in Fig. 5b). However, due to preferential wetting, horizontal A-B-A and B-A-B lamellae form on the stripe and substrate, respectively (in contrast, in Fig. 5b, horizontal B-A-B and A-B-A lamellae initially form on the stripe and substrate, respectively). The energetically unfavorable contact of A with the free surface only exists above the relatively narrow stripe (in contrast, in Fig. 5b, this unfavorable contact exists above the relatively expansive substrate). To minimize this small area of unfavorable contact, BCP from horizontal lamellae above the substrate transfers to the stripe to create horizontal A-B-A-B lamellae on the stripe. Consequently, the final BCP morphology consists of horizontal lamellae, in which B is in contact with the free surface, on both the stripe and substrate (Supplementary Fig. 25). In contrast, a large amount of BCP is transferred in Fig. 5 to minimize the large area of unfavorable contact, resulting in formation of slanted lamellae at the stripe/substrate boundaries (Fig. 5e). These slanted lamellae subsequently drive formation of vertical lamellae on the stripe, resulting in the final BCP morphology observed in boundary-directed epitaxy (Fig. 5f).

Effect of block copolymer incompressibility on boundary-directed epitaxy. The effect of the surface energy difference between A and B on boundary-directed epitaxy is explored by varying $\kappa_A N$ from 150 to 250 while $\kappa_B N$ is constant at 100. Larger values of $\kappa_A N$ indicate a larger surface tension of A, which results in a stronger preferentiality for B to wet the free surface. Boundary-directed epitaxy is achieved when $\kappa_A N$ is varied from 170 to 230 (Fig. 5f). When the surface energy difference is too small (e.g., $\kappa_A N = 150$), the driving force to induce local mass transfer is relatively weak. Consequently, horizontal lamellae that are out of phase at the stripe/substrate boundaries (like in Fig. 5b) are stabilized on both the stripe and substrate (Supplementary Fig. 24a). In contrast, when the surface energy difference is too large (e.g., $\kappa_A N = 250$), contact of A with the free surface is highly unfavorable, inhibiting formation of slanted lamellae at the stripe/substrate boundaries (see Fig. 5e). In this case, horizontal lamellae form on both the stripe and substrate, forming holes/islands in which B is in contact with the free surface (Supplementary Fig. 24c).

Effect of block copolymer film thickness on boundary-directed epitaxy. Boundary-directed epitaxy is achieved when the initial relaxed BCP film thickness (i.e., the BCP thickness after a few MC cycles) is varied from $1.3L_0$ (Supplementary Fig. 26a) to $0.95L_0$ (Supplementary Fig. 26b). When the initial relaxed BCP film thickness is reduced to $0.8L_0$, boundary-directed epitaxy is not observed (Supplementary Fig. 26c,d). Instead, initially, horizontal A-B and A-B-A lamellae form on the substrate, while horizontal B-A-

B lamellae form on the stripe (Supplementary Fig. 26c). As discussed above, to minimize the energetically unfavorable contact of A with the free surface, the BCP flows to form horizontal A-B-A-B lamellae on the substrate (Supplementary Fig. 26d). However, BCP can flow to the horizontal A-B-A lamellae both from horizontal A-B lamellae on the substrate, as well as from horizontal B-A-B lamellae on the stripe. Thus, directional BCP flow from stripe to substrate does not occur, slanted lamellae (see Fig. 5e) do not form, and the BCP morphology after boundary-directed epitaxy (see Fig. 5f) is not observed. It is possible that given sufficient time, however, the island at the left edge of the stripe would diffuse away from the stripe/substrate boundary, slanted lamellae would be induced, vertical lamellae would be nucleated on the stripe, and boundary-directed epitaxy would be realized.

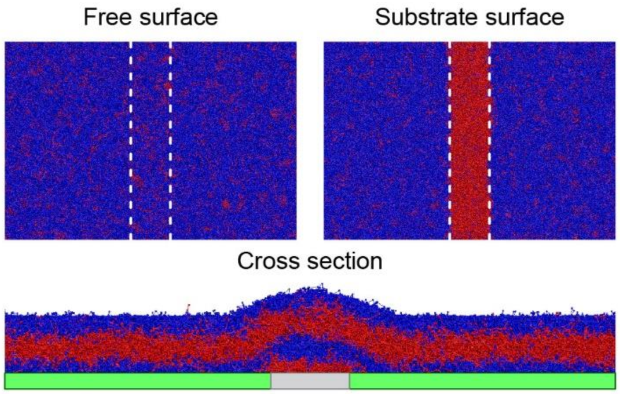

Supplementary Fig. 25 Monte Carlo simulations on templates in which the stripe and substrate materials are exchanged. Simulated BCP morphology at the free surface (top left), at the substrate surface (top right), and along a cross-section perpendicular to the stripe width (bottom) on a stripe with width of $1.5L_0$ for $\lambda_{\text{B-stripe}}N$, $\lambda_{\text{B-substrate}}N$, $\lambda_{\text{A-stripe}}N$, and $\lambda_{\text{A-substrate}}N$ of -2.0, -2.35, -2.5 and -2.0, respectively, which represents a Ge stripe on a graphene substrate. The stripe and the substrate materials are exchanged with respect to those in Fig. 5. The stripe, substrate, B, and A are grey, green, blue, and red, respectively. White dashed lines indicate the positions of the stripe/substrate boundaries.

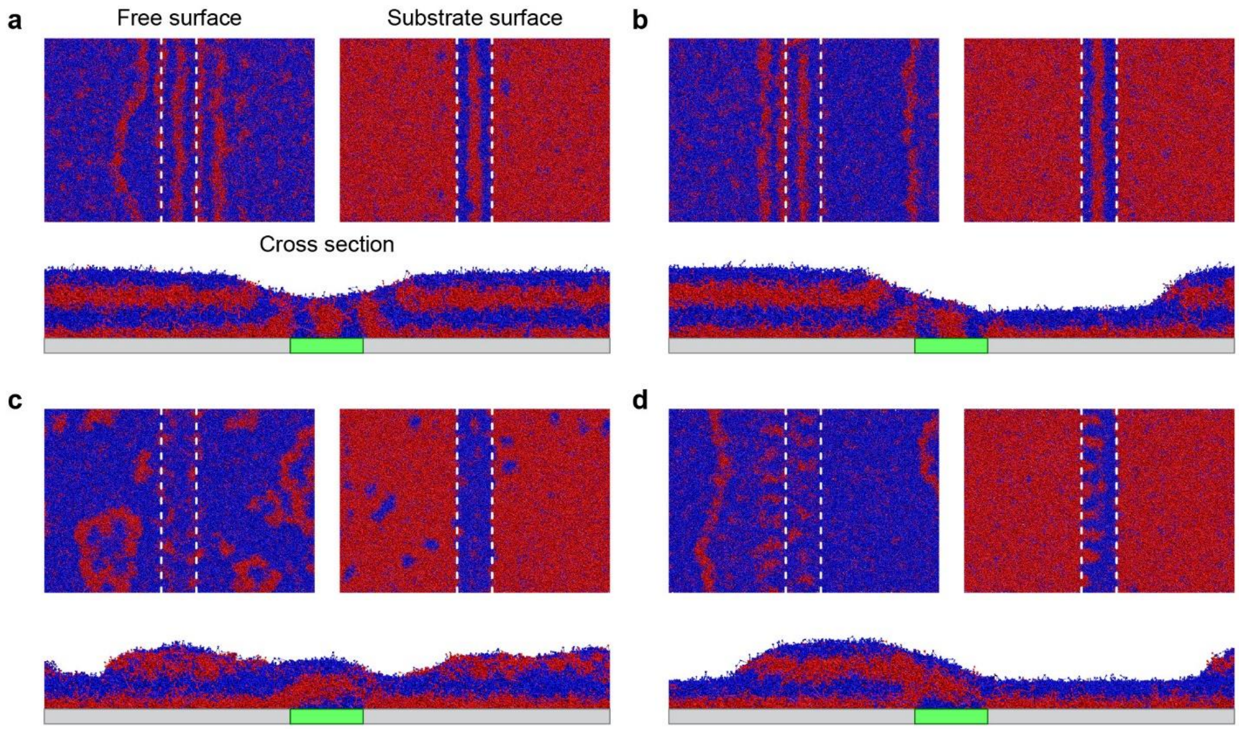

Supplementary Fig. 26 Monte Carlo simulations with different initial BCP film thicknesses. a-d, Simulated BCP morphology at the free surface (top left), at the substrate surface (top right), and along a cross-section perpendicular to the stripe width (bottom) on a stripe with width of $1.5L_0$ for an initial relaxed BCP film thickness of $1.3L_0$ (**a**), $0.95L_0$ (**b**), and $0.8L_0$ (**c,d**). In contrast, in Fig. 5, the initial relaxed BCP film thickness is $1.15L_0$. For the initial relaxed BCP film thickness of $0.8L_0$, assembly is shown after 5×10^4 (**c**) and 2×10^6 (**d**) MC cycles. The stripe, substrate, B, and A are green, grey, blue, and red, respectively. White dashed lines indicate the positions of the stripe/substrate boundaries.

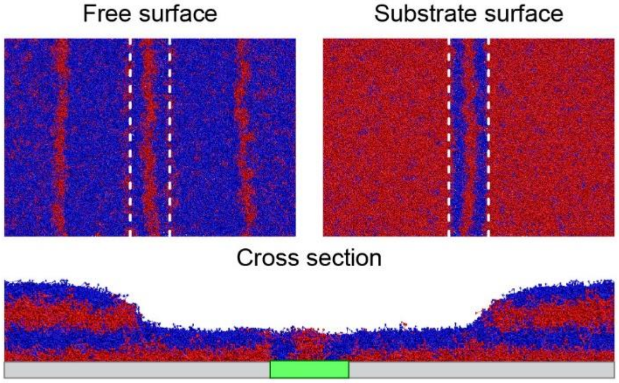

Supplementary Fig. 27 Monte Carlo simulations of boundary-directed epitaxy with a 3.4 Å step height at the stripe/substrate boundaries. Simulated BCP morphology at the free surface (top left), at the substrate surface (top right), and along a cross-section perpendicular to the stripe width (bottom) on a stripe with width of $1.5L_0$ and thickness of $0.027L_0$. The stripe thickness of $0.027L_0$ represents the experimental step height at the stripe/substrate boundaries of 3.4 Å (i.e., the thickness of monolayer graphene). The BCP morphology matches that in Fig. 5f, in which there is zero topography at the boundaries and the surface of the template is perfectly flat. The stripe, substrate, B, and A are green, grey, blue, and red, respectively. White dashed lines indicate the positions of the stripe/substrate boundaries.

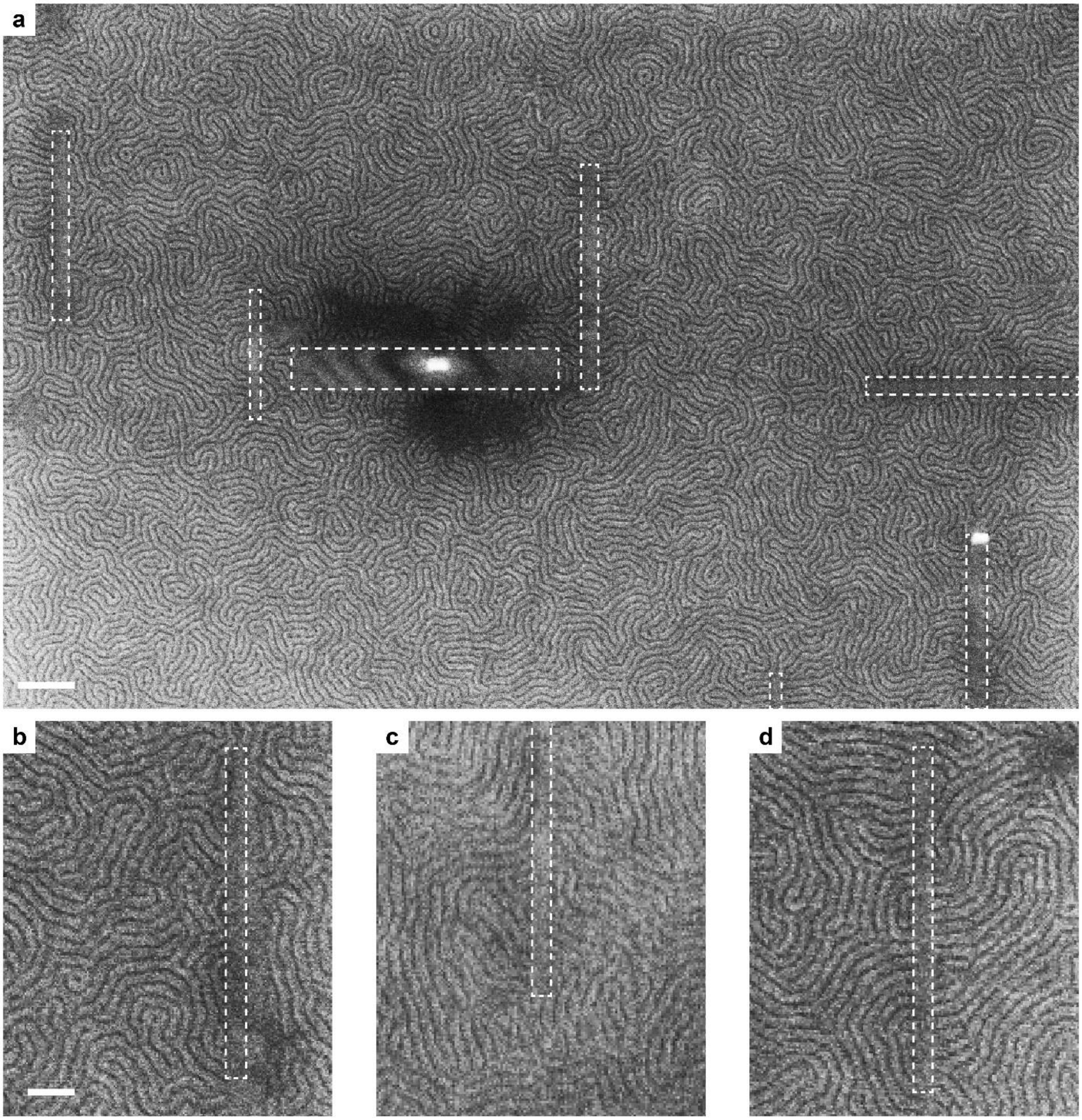

Supplementary Fig. 28 Assembly of PS-b-PMMA on graphene stripes. a-d, Assembly of PS-b-PMMA (L_0 of 25 nm) with film thickness of ~L_0 on graphene stripes with various widths (**a**) and widths of 1.5L_0 (**b-d**). The samples are annealed at 160 (**a**,**b**), 190 (**c**), and 220 °C (**d**) for 10 min. Scale bars are 200 (**a**) and 100 nm (**b-d**). Small disordered grains of vertical lamellae (i.e., a fingerprint pattern) are formed on the Ge background and horizontal lamellae are formed on the graphene stripes. Graphene is preferential to PS but Ge is neutral to PS-b-PMMA[29].

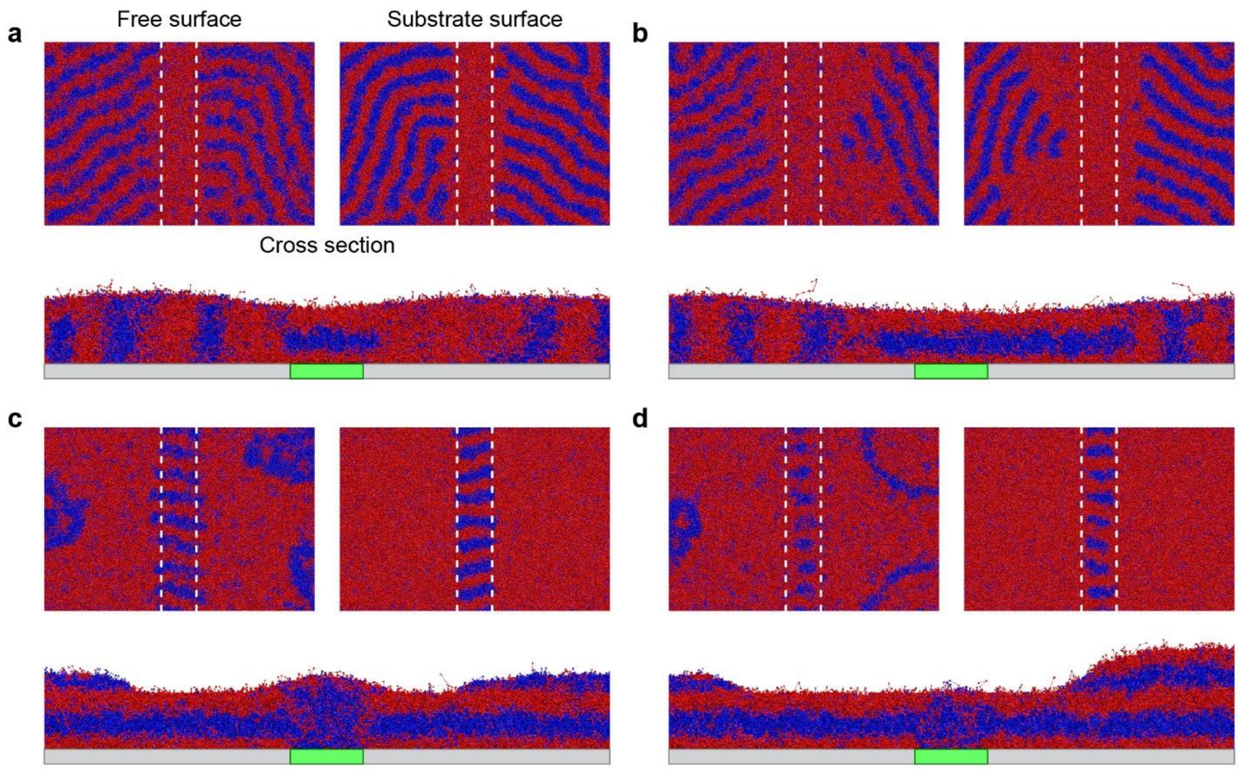

Supplementary Fig. 29 Monte Carlo simulations on chemical patterns with different guiding schemes. **a**-**d**, Simulated BCP morphology at the free surface (top left), at the substrate surface (top right), and along a cross-section perpendicular to the stripe width (bottom) after 2.5 × 10^5 (**a**,**c**) and 3 × 10^6 (**b**,**d**) MC cycles on a stripe with width of 1.5L_0. Simulations are performed for two Guiding Schemes: (1) the stripe is preferential to A and the background is neutral (**a**,**b**) and (2) the stripe is neutral and the background is preferential to A (**c**,**d**). In contrast, a third Guiding Scheme is used in Fig. 5, in which the stripe is preferential to B and the background is preferential to A. The stripe, substrate, B, and A are green, grey, blue, and red, respectively. After 2.5 × 10^5 MC cycles in Guiding Scheme 1, horizontal lamellae form on the stripe, whereas vertical lamellae in a fingerprint pattern form on the background (**a**). As assembly evolves after 3 × 10^6 MC cycles, the horizontal lamellae expand further onto the background, reducing the percentage of the surface covered by the fingerprint pattern (**b**). After 2.5 × 10^5 MC cycles in Guiding Scheme 2, vertical lamellae form on the stripe and align perpendicular to the stripe edge, whereas horizontal lamellae form on the background (**c**). As assembly evolves after 3 × 10^6 MC cycles, the length of the vertical lamellae decreases as BCP diffuses away from the stripe surface and onto the background (**d**).

Supplementary references

1. Suh, H. S., Kang, H. M., Nealey, P. F. & Char, K. Thickness dependence of neutral parameter windows for perpendicularly oriented block copolymer thin films. *Macromolecules* **43,** 4744-4751 (2010).
2. McElhinny, K. M., Jacobberger, R. M., Zaug, A. J., Arnold, M. S. & Evans, P. G. Graphene-induced Ge (001) surface faceting. *Surf. Sci.* **647,** 90-95 (2016).
3. Jacobberger, R. M. et al. Direct oriented growth of armchair graphene nanoribbons on germanium. *Nat. Commun.* **6,** 8006 (2015).
4. Dann, J. R. Forces involved in the adhesive process: I. critical surface tensions of polymeric solids as determined with polar liquids. *J. Colloid Interface Sci.* **32,** 302-320 (1970).
5. Wu, S. Surface and interfacial tensions of polymer melts. II. poly(methyl methacrylate), poly(n-butyl methacrylate), and polystyrene. *J. Phys. Chem.* **74,** 632-638 (1970).

6. Wu, S. Calculation of interfacial tension in polymer systems. *J. Polym. Sci., Part C: Polym. Symp.* **34,** 19-30 (1971).
7. Kim, G. et al. Biological affinity and biodegradability of poly(propylene carbonate) prepared from copolymerization of carbon dioxide with propylene oxide. *Macromol. Res.* **16,** 473-480 (2008).
8. Way, A. J., Jacobberger, R. M. & Arnold, M. S. Seed-initiated anisotropic growth of unidirectional armchair graphene nanoribbon arrays on germanium. *Nano Lett.* **18,** 898-906 (2018).
9. Way, A. J. et al. Anisotropic synthesis of armchair graphene nanoribbon arrays from sub-5 nm seeds at variable pitches on germanium. *J. Phys. Chem. Lett.* **10,** 4266-4272 (2019).
10. Stoykovich, M. P. et al. Directed self-assembly of block copolymers for nanolithography: fabrication of isolated features and essential integrated circuit geometries. *ACS Nano* **1,** 168-175 (2007).
11. Detcheverry, F. A., Pike, D. Q., Nealey, P. F., Müller, M. & de Pablo, J. J. Monte Carlo simulation of coarse grain polymeric systems. *Phys. Rev. Lett.* **102,** 197801 (2009).
12. Detcheverry, F. A., Pike, D. Q., Nagpal, U., Nealey, P. F. & de Pablo, J. J. Theoretically informed coarse grain simulations of block copolymer melts: method and applications. *Soft Matter* **5,** 4858-4865 (2009).
13. Detcheverry, F. A., Liu, G., Nealey, P. F. & de Pablo, J. J. Interpolation in the directed assembly of block copolymers on nanopatterned substrates: simulation and experiments. *Macromolecules* **43,** 3446-3454 (2010).
14. Detcheverry, F. A. et al. Monte Carlo simulations of a coarse grain model for block copolymers and nanocomposites. *Macromolecules* **41,** 4989-5001 (2008).
15. Hömberg, M. & Müller, M. Main phase transition in lipid bilayers: phase coexistence and line tension in a soft, solvent-free, coarse-grained model. *J. Chem. Phys.* **132,** 155104 (2010).
16. Hur, S.-M. et al. Simulation of defect reduction in block copolymer thin films by solvent annealing. *ACS Macro Lett.* **4,** 11-15 (2015).
17. Wang, J. & Müller, M. Microphase separation of mixed polymer brushes: dependence of the morphology on grafting density, composition, chain-length asymmetry, solvent quality, and selectivity. *J. Phys. Chem. B* **113,** 11384-11402 (2009).
18. Müller, M. Phase diagram of a mixed polymer brush. *Phys. Rev. E* **65,** 030802 (2002).
19. Müller, M. & Smith, G. D. Phase separation in binary mixtures containing polymers: a quantitative comparison of single-chain-in-mean-field simulations and computer simulations of the corresponding multichain systems. *J. Polym. Sci., Part B: Polym. Phys.* **43,** 934-958 (2005).
20. Hur, S.-M. et al. Interplay of surface energy and bulk thermodynamic forces in ordered block copolymer droplets. *Macromolecules* **48,** 4717-4723 (2015).
21. Matsen, M. W. & Thompson, R. B. Equilibrium behavior of symmetric ABA triblock copolymer melts. *J. Chem. Phys.* **111,** 7139-7146 (1999).
22. Onses, M. S. et al. Block copolymer assembly on nanoscale patterns of polymer brushes formed by electrohydrodynamic jet printing. *ACS Nano* **8,** 6606-6613 (2014).
23. Lu, W. & Ding, J. Dynamic Monte Carlo simulation of polymerization of amphiphilic macromers in a selective solvent and associated chemical gelation. *Macromolecules* **39,** 7433-7440 (2006).
24. Sanz, E. & Marenduzzo, D. Dynamic Monte Carlo versus Brownian dynamics: a comparison for self-diffusion and crystallization in colloidal fluids. *J. Chem. Phys.* **132,** 194102 (2010).
25. Filion, L., Hermes, M., Ni, R. & Dijkstra, M. Crystal nucleation of hard spheres using molecular dynamics, umbrella sampling, and forward flux sampling: a comparison of simulation techniques. *J. Chem. Phys.* **133,** 244115 (2010).
26. Stasiak, P., McGraw, J. D., Dalnoki-Veress, K. & Matsen, M. W. Step edges in thin films of lamellar-forming diblock copolymer. *Macromolecules* **45,** 9531-9538 (2012).
27. Kim, J. U. & Matsen, M. W. Droplets of structured fluid on a flat substrate. *Soft Matter* **5,** 2889-2895 (2009).
28. Shin, D. O. et al. One-dimensional nanoassembly of block copolymers tailored by chemically patterned surfaces. *Macromolecules* **42,** 1189-1193 (2009).

29. Chang, T.-H. et al. Directed self-assembly of block copolymer films on atomically-thin graphene chemical patterns. *Sci. Rep.* **6,** 31407 (2016).
